# Biological characteristics of tissue engineered-nerve grafts enhancing peripheral nerve regeneration

**DOI:** 10.1186/s13287-024-03827-9

**Published:** 2024-07-18

**Authors:** Xiangling Li, Hang Xu, Chaochao Li, Yanjun Guan, Yuli Liu, Tieyuan Zhang, Fanqi Meng, Haofeng Cheng, Xiangyu Song, Zhibo Jia, Ruichao He, Jinjuan Zhao, Shengfeng Chen, Congcong Guan, Shi Yan, Jinpeng Wang, Yu Wei, Jian Zhang, Jinshu Tang, Jiang Peng, Yu Wang

**Affiliations:** 1 The Fourth Medical Center of the General Hospital of People’s Liberation Army, Beijing, 100853 China; 2 Department of Rehabilitation, Tongji Hospital, Tongji Medical College, Huazhong University of Science and Technology, Wuhan, China; 3 Department of General Surgery, General Hospital, Tianjin Medical University, Tianjin, 300052 China; 4 Institute of Orthopedics, Chinese PLA General Hospital, Beijing Key Lab of Regenerative Medicine in Orthopedics, Key Laboratory of Musculoskeletal Trauma & War Injuries PLA, Beijing, 100853 China; 5 Co-Innovation Center of Neuroregeneration, Nantong University, Nantong, 226001 China; 6 School of Medicine, Nankai University, Tianjin, 300071 China; 7 School of Medicine, Hebei North University, Zhangjiakou, 075132 China; 8 Shandong University Center for Orthopaedics, Cheeloo College of Medicine, Shandong University, Jinan, 250012 China; 9 Department of Anesthesiology, Xuanwu Hospital Capital Medical University, Beijing, 100053 China

**Keywords:** Peripheral nerve injury, Extracellular matrix, Mesenchymal stem cells, Vascular regeneration, Whole transcriptome sequencing

## Abstract

**Background:**

A favorable regenerative microenvironment is essential for peripheral nerve regeneration. Neural tissue-specific extracellular matrix (ECM) is a natural material that helps direct cell behavior and promote axon regeneration. Both bone marrow-derived mesenchymal stem cells (BMSCs) and adipose-derived mesenchymal stem cells (ADSCs) transplantation are effective in repairing peripheral nerve injury (PNI). However, there is no study that characterizes the in vivo microenvironmental characteristics of these two MSCs for the early repair of PNI when combined with neural tissue-derived ECM materials, i.e., acellular nerve allograft (ANA).

**Methods:**

In order to investigate biological characteristics, molecular mechanisms of early stage, and effectiveness of ADSCs- or BMSCs-injected into ANA for repairing PNI in vivo, a rat 10 mm long sciatic nerve defect model was used. We isolated primary BMSCs and ADSCs from bone marrow and adipose tissue, respectively. First, to investigate the in vivo response characteristics and underlying molecular mechanisms of ANA combined with BMSCs or ADSCs, eighty-four rats were randomly divided into three groups: ANA group, ANA+BMSC group, and ANA+ADSC group. We performed flow cytometry, RT-PCR, and immunofluorescence staining up to 4 weeks postoperatively. To further elucidate the underlying molecular mechanisms, changes in long noncoding RNAs (lncRNAs), circular RNAs (circRNAs), microRNAs (miRNAs), and messenger RNAs (mRNAs) were systematically investigated using whole transcriptome sequencing. We then constructed protein–protein interaction networks to find 10 top ranked hub genes among differentially expressed mRNAs. Second, in order to explore the effectiveness of BMSCs and ADSCs on neural tissue-derived ECM materials for repairing PNI, sixty-eight rats were randomized into four groups: ANA group, ANA+BMSC group, ANA+ADSC group, and AUTO group. In the ANA+BMSC and ANA+ADSC groups, ADSCs/BMSCs were equally injected along the long axis of the 10-mm ANA. Then, we performed histological and functional assessments up to 12 weeks postoperatively.

**Results:**

The results of flow cytometry and RT-PCR showed that ANA combined with BMSCs exhibited more significant immunomodulatory effects, as evidenced by the up-regulation of interleukin (IL)-10, down-regulation of IL-1β and tumor necrosis factor-alpha (TNF-α) expression, promotion of M1-type macrophage polarization to M2-type, and a significant increase in the number of regulatory T cells (Tregs). ANA combined with ADSCs exhibited more pronounced features of pro-myelination and angiogenesis, as evidenced by the up-regulation of myelin-associated protein gene (MBP and MPZ) and angiogenesis-related factors (TGF-β, VEGF). Moreover, differentially expressed genes from whole transcriptome sequencing results further indicated that ANA loaded with BMSCs exhibited notable immunomodulatory effects and ANA loaded with ADSCs was more associated with angiogenesis, axonal growth, and myelin formation. Notably, ANA infused with BMSCs or ADSCs enhanced peripheral nerve regeneration and motor function recovery with no statistically significant differences.

**Conclusions:**

This study revealed that both ANA combined with BMSCs and ADSCs enhance peripheral nerve regeneration and motor function recovery, but their biological characteristics (mainly including immunomodulatory effects, pro-vascular regenerative effects, and pro-myelin regenerative effects) and underlying molecular mechanisms in the process of repairing PNI in vivo are different, providing new insights into MSC therapy for peripheral nerve injury and its clinical translation.

**Supplementary Information:**

The online version contains supplementary material available at 10.1186/s13287-024-03827-9.

## Background

Peripheral nerve injuries (PNIs) account for approximately 1.5–4.0% of all trauma cases worldwide and have a poor postoperative prognosis, often leading to motor dysfunction, sensory abnormalities and pain syndromes in patients [1–3]. Although autologous nerve grafts are considered the gold standard for clinical treatment, some shortcomings remain [4]. Peripheral nerve regeneration is a multifaceted process involving the regeneration of blood vessels, axons, and myelin sheaths. It also involves the modulation of inflammatory and regenerative factors, as well as the synthesis and release of neurotransmitters [5, 6]. Among them, Schwann cells (SCs), endothelial cells, and immune cells such as macrophages, neutrophils, and lymphocytes all play important roles and participate in the formation of a good local regenerative microenvironment [5, 6]. Allogeneic extracellular matrix (ECM)-derived graft materials are considered to be one of the effective alternatives to autologous nerve grafts for repairing nerve defects, which not only avoids immune rejection that occurs after transplantation but also preserves the ECM components and biomechanical properties, which have been proved by multiple studies to be effective in promoting nerve regeneration [7–10], and can be used to guide the cellular migration and axonal growth trajectories [11], as well as stimulating axonal and myelin regeneration of SCs and promoting stem cell proliferation and differentiation into neurons [11–13]. The ECM is a unique and tissue-specific natural material that contains a diverse array of growth factors, including platelet-derived growth factor (PDGF), epidermal growth factor receptor (EGFR), and vascular endothelial growth factor receptor (VEGFR). It is also rich in structural and functional proteins, such as fibronectin, elastin, collagen, glycoproteins (e.g., proteoglycans, fibronectin, laminin), among others [14]. And the ECM serves various crucial functions, including binding growth factors, transmitting cell signals, facilitating cell adhesion, regulating pH in the microenvironment, participating in tissue differentiation, and influencing cell behavior during processes like migration, differentiation, adhesion, proliferation, and apoptosis, as well as directing intrinsic cellular phenotype and survival [11]. Due to the lack of cellular active components in the allogenic ECM-derived graft material, its effectiveness in repairing long segmental PNI warrants further improvement.

Currently, cellular therapy is one of the most innovative therapeutic approaches in the field of nerve repair [15]. Particularly, transplantation of mesenchymal stem cells (MSCs) is effective in promoting peripheral nerve regeneration, with a wide range of sources, possessing the plasticity of differentiation toward neural cells, which can replace damaged tissue cells, secreting neurotrophic and vascular regeneration factors, and possessing a good immune-modulating effect to improve the intrinsic regenerative capacity of the tissues [16]. Compared with other types of MSCs, bone marrow-derived mesenchymal stem cells (BMSCs) and adipose-derived mesenchymal stem cells (ADSCs) are the most commonly used MSCs, with higher paracrine factor production, and they also possess the advantages of being easily accessible, easily cultivable, low immunogenicity, and from a wide range of sources [17]. Both BMSCs and ADSCs have demonstrated strong therapeutic effects in peripheral nerve injury models [17, 18]. BMSCs exhibit neuroprotective and regenerative properties, including promoting axonal growth and vascular regeneration [18], reducing neuronal apoptosis [19], and facilitating homing and differentiation of endogenous stem cells [20, 21]. Similarly, ADSCs have been shown to improve sensory and motor function, promote axonal regeneration, and enhance myelin production through glial cell line-derived neurotrophic factor (GDNF) signaling [18].

A comparative analysis of BMSCs with ADSCs indicated that there is no significant difference between the two in terms of morphological and phenotypic characteristics [22], and both can be induced to become early and mature neural ectodermal cells using specific methods [23, 24]. In vitro nerve growth factor (NGF) and brain-derived neurotrophic factor (BDNF) secretion functions of ADSCs are also stronger than those of BMSCs, and ADSCs have more potential than BMSCs to repair nerve damage [25]. Lavorato et al. [26] summarized the main characteristics of different stem cells and inferred that ADSCs are the most suitable MSCs for repairing PNI. However, there is no study that characterizes the in vivo microenvironmental characteristics of these two MSCs for the early repair of PNI when combined with neural tissue-derived ECM materials, i.e. acellular nerve allograft (ANA). Therefore, the aim of this study is to find out the microenvironmental characteristics of these two MSCs for the early repair of PNI, which will help to provide a theoretical basis for selecting the most appropriate MSCs for different patients in the future clinical applications. We primarily used ANA, which mimic the natural structure and compositional microenvironment of the ECM and support neural regeneration, influence cellular bioactivity and differentiation function, and ensure a homogeneous distribution of MSCs [27, 28]. And we used a model of sciatic nerve defect injury in rats in order to investigate the early repair characteristics and related molecular mechanisms of neural tissue-derived ECM materials composite with BMSCs and ADSCs, respectively.

## Materials and methods

### Preparation of acellular nerve allograft

68 Sprague–Dawley (SD) rats (6–8 weeks old, 220–250 g) were sacrificed after anesthetized by intraperitoneal injection of an overdose of 3% sodium pentobarbital, then the bilateral sciatic nerve segments (20–30 mm) were collected. Fat and connective tissues were removed and decellularized based on previous methods with some modifications [29, 30]. Nerve segments were immersed in double-distilled water (ddH2O) for 12 h, then rinsed thrice in phosphate-buffered saline (PBS), re-immersed in ddH2O, frozen at − 80 °C for 12 h. After thawing, the nerve segments were treated with 1% sodium dodecyl sulfate (SDS) (Sigma, USA) for 24 h at room temperature on a shaker at 60 rpm, then were repeatedly washed with PBS to remove SDS until the PBS wash solution became clear and no foam was generated. ANA were subsequently sealed and stored in PBS containing 100 U/mL of penicillin and streptomycin, followed by sterilization using Co^60^ gamma radiation for immediate use or stored at − 20 °C for later use.

### DNA quantification

To measure the remaining DNA in decellularized nerves, we lyophilized fresh and decellularized sciatic nerves (n = 3) from rats, then weighed them and extracted the total DNA using a nucleic acid extraction analyzer. Next, the fluorescence intensity values were read on a fluorescence spectrophotometer at an excitation wavelength of 480 nm and an emission wavelength of 520 nm using a quantitative nucleic acid detection kit (Invitrogen, P7589). The standard curve was then plotted and the DNA content was calculated.

### Scanning electron microscopy (SEM)

Normal and decellularized nerves were dehydrated using graded ethanol solutions at concentrations of 30, 50, 70, 85, 95, 100, and 100% for 10 min each. After dehydration, the nerves were dried with a CO_2_ extraction dryer. The coating was then photographed using SEM (6700; JEOL).

### Extraction of primary ADSCs

After five 3-day-old SD rats were euthanized, the adipose tissue was collected by dissecting the inguinal region of and immediately washed with PBS. After washing off the blood, the adipose tissue was minced with microscopic scissors and then digested with 0.1% (W/V) type I collagenase (Sigma-Aldrich, USA) for 40 min in a 37 °C environment. Complete Dulbecco’s Modified Eagle Medium (DMEM, Gibco, USA) supplemented with 100 U/ml penicillin/streptomycin and 10% fetal bovine serum was added to terminate digestion. The tissue debris was subsequently filtered using a 100 μm nylon mesh and re-filtered using a 40 μm nylon mesh. The cell suspension was centrifuged at 1500 rpm for 5 min and the supernatant was discarded. The cell pellet was resuspended in a complete DMEM medium, seeded in 25 cm^2^ culture flasks, and then cultured at 37 °C in a 5% CO_2_ atmosphere. The solution was changed after 48 h. After reaching 80% confluence, cells were passaged and P2-P4 generations were used for subsequent analysis.

### Extraction of primary BMSCs

After five 3-day-old SD rats were euthanized, the femur and tibia of rats were isolated bilaterally under aseptic conditions, washed twice in PBS, both epiphyses were clipped, and fresh bone marrow was flushed out using a 1 mL syringe aspirated with complete DMEM medium containing 100 U/ml penicillin/streptomycin and 10% fetal bovine serum (Gibco, USA). After centrifugation at 1500 rpm for 5 min, the supernatant was discarded. The cell pellet was resuspended in a complete DMEM medium, seeded in 25 cm^2^ culture flasks, and incubated in an incubator at 37 °C in a 5% CO_2_ atmosphere. The solution was changed after 24 h. After reaching 80% confluence, cells were passaged and P2-P4 generations were used for subsequent analysis.

### Identification of MSCs by flow cytometry

Both MSCs were passaged to P2 generation, digested using 0.25% trypsin, a cell volume of 5 × 10^5^ was collected from each, and cells were resuspended in phosphate PBS buffer (Corning, 21-031-CV) for subsequent analysis. Fluorescein isothiocyanate (FITC) anti-human CD90 (BioLegend, 328107, USA), phycoerythrin (PE)/Cyanine7 anti-human leukocyte antigen (HLA) -DR (BioLegend, 307105, USA), allophycocyanin (APC) anti-human CD73 (BioLegend, 344005, USA), PE anti-CD34 (BioLegend, 343605, USA), PerCP anti-human CD45 (BioLegend, 368505, USA), and Brilliant violet421 anti-CD105 (BioLegend, 800509, USA) antibodies were incubated for 15 min in the dark. Subsequent characterization was performed with the DxFLEX flow cytometer using the FlowJo v.7,6.5 software.

### Animal model

#### Sciatic nerve defect modeling and bridging surgery

The work has been reported in line with the ARRIVE guidelines 2.0. Female SD rats (6–8 weeks old, 220–250 g) were anesthetized by intraperitoneal injection of 3% sodium pentobarbital (30 mg/kg). Next, the right hind limb was prepared and sterilized, and the skin and musculature of the right lateral thigh were incised on a sterile operating table to expose and cut off the sciatic nerve to create a 10 mm gap. The experiment was divided into two parts. First, to investigate the in vivo response characteristics and underlying molecular mechanisms of ANA combined with BMSCs or ADSCs, eighty-four rats were randomly divided into three groups (n = 28 rats per group): ANA group, ANA+BMSC group, and ANA+ADSC group. Second, in order to explore the effectiveness of BMSCs and ADSCs on neural tissue-derived ECM materials for repairing PNI, sixty-eight rats were randomized into four groups (n = 17 rats per group): ANA group, ANA+BMSC group, ANA+ADSC group, and AUTO group. In the ANA+BMSC and ANA+ADSC groups, 25 μL ADSCs/BMSCs containing 2 × 10^6^/ml (suspended in DMEM) was equally injected into ANA along the long axis of the 10-mm ANA using a 31G insulin syringe needle. The insulin syringe was pushed at a constant speed in 1 min to fill the entire ANA with the suspensions of BMSCs and ADSCs, respectively, and the grafts (i.e., ANA loaded with BMSCs/ADSCs) were then sutured to the nerve ends. In the AUTO group, the dissected 10-mm sciatic nerves were turned 180° and sutured. The nerve was sutured using a 10–0 tension-free band sutures. Subsequently, the muscle and skin were subsequently sutured layer-by-layer using 4–0 sutures. All rats were sterilized with iodophor to disinfect the wounds and then housed in a 12-h light/dark cycle, with food and water ad libitum for the entire study duration.

#### Immunofluorescence staining

To evaluate assess the degree of decellularization and characterize the composition of the extracellular matrix, the collected ANA were fixed in 4% paraformaldehyde (PFA) solution for 24 h and dehydrated using 30% sucrose solution. They were then embedded and sliced longitudinally into a thickness of 9 μm in a cryostat. The slices were washed three times with PBS and then incubated with 10% goat sealing serum for 40 min. Rabbit anti-Fibronectin (1:100, Abcam, ab2413) was used as a primary antibody, and nuclear staining was performed using 4’,6-diamidino-2-phenylindole (DAPI) (BOSTER, AR1176).

At 2 and 3 weeks postoperatively, the rats were executed by overdose of anesthesia, and the nerve grafts in each group were removed for fixation, dehydration, and slicing in the same procedure as described above. Slices were washed thrice with PBS, permeabilized with 0.5% Triton X-100 (Sigma) for 10 min, and then blocked for 40 min. Sections were incubated in a refrigerator at 4 °C overnight with primary antibodies, including mouse anti-neurofilament 200 (NF200) (1:400, Sigma, N0142), rabbit anti-S100β (1:150, Abcam, ab52642), rabbit anti-myelin protein zero (MPZ) (1:50, Abcam, ab183868), mouse anti-alpha-smooth muscle actin (α-SMA) (1:400, Abcam, ab7817), and rabbit anti-CD31 (1:100, Abcam, ab222783), and rewarmed at room temperature for 30 min on the following day. Then, sections were incubated with secondary antibodies, including goat anti-rabbit-Alexa Fluor®594 (1:200, Abcam, ab150080) and goat anti-mouse-Alexa Fluor®488 (1:200, Abcam, ab150117) for 2 h at room temperature. The nuclei were stained with DAPI for 10 min, and then observed and photographed under the fluorescence microscope (Nikon, Tokyo) and Pannoramic Confocal panoramic scanner (3DHISTECH).

#### Real-time quantitative polymerase chain reaction (RT-PCR) analysis

Briefly, each frozen nerve tissue was ground into a powder using liquid nitrogen, and total RNA was extracted using TRIzol (Ambion, USA). ReverTra Ace qPCR RT premix (TOYOBO, FSQ-201, Japan) was used to reverse-transcribe RNA to complementary DNA (cDNA). Subsequently, RT-PCR was performed on a StepOnePlus TM RT PCR System (Roche Diagnostics, US) using the RT2 SYBR Green qPCR Mastermix (GenStar, A303-10). The 2^−ΔΔCT^ method was used to determine nerve regeneration-related genes (MBP, MPZ, and c-Jun), neurotrophic factor-related genes (BDNF, NGF, neurotrophin-3(NT-3)), and inflammation-related genes (IL-1β, IL-6, TNF-α, IL-10, IL-13, IL-4, inducible nitric oxide synthase (iNOS), CD206, arginase-1 (ARG-1)) at their relative expression levels. All primers were obtained from Biogenesis and their specific sequences are listed in Table 1.

**Table 1:** Primer sequences used in RT-quantitative polymerase chain reaction studies

Target genes	Forward primer (5’-3’)	Reverse primer (3’-5’)
GAPDH	ACAGCAACAGGGTGGTGGAC	TTTGAGGGTGCAGCGAACTT
VEGF	GGCTCACTTCCAGAAACACG	GTGCTCTTGCAGAATCTAGTGG
IL-6	TCTGCTCTGGTCTTCTGGAGTTCC	GAGTTGGATGGTCTTGGTCCTTAGC
IL-1β	CGACCTGCTAGTGTGTGATGTTCC	GGTGGGTGTGCCGTCTTTCATC
Arg-1	CATATCTGCCAAGGACATCGT	TCCATCACTTTGCCAATTCCC
NGF	AAGGACGCAGCTTTCTATCC	CTATCTGTGTACGGTTCTGCC
iNOS	AGGCACAAGACTCTGACACCC	CGCACTTCTGTCTCTCCAAACCC
CD206	TGTTTTGGCTGGGACTGACCTA	CGGGTGTAGGCTCGGGTAGTAG
BDNF	AAGTCTGCATTACATTCCTCGA	GTTTTCTGAAAGAGGGACAGTTTAT
IL-4	CAAGGAACACCACGGAGAACGAG	CTTCAAGCACGGAGGTACATCACG
IL-13	CTCGCTTGCCTTGGTGGTCTTG	GCACAGGGAAGTCTTCTGGTCTTG
IL-10	CCAAGCCTTATCGGAAATGA	TTTTCACAGGGGAGAAATCG
TGF-β	GACCGCAACAACGCAATCTATGAC	CTGGCACTGCTTCCCGAATGTC
TNF-α	CGTCAGCCGATTTGCTATCT	CGGACTCCGCAAAGTCTAAG
c-Jun	GACCTTCTACGACGATGC	CAGCGCCAGCTACTGAGGC
MBP	CGCATCTTGTTAATCCGTTCTAAT	GAGGGTTTGTTTCTGGAAGTTTC
MPZ	CATTGTGGTTTACACGGACAG	CTTGGCATAGTGGAAGATTGA

#### Flow cytometry analysis

At 1 and 2 weeks postoperatively, nerve grafts in each group were obtained under aseptic conditions, sliced with scissors, and transferred to a mixture of collagenase I (0.5 mg/ml, C8490, Solarbio), collagenase II (0.5 mg/mL, C8150, Solarbio), and DNase I (25 µg/mL, D8070, Solarbio), followed by the addition of Hank’s Balanced Salt Solution (HBSS) for dissociation and incubation at 37 °C for 35 min with stirring every 5 min. Immune cells were stained intracellularly and on the cell surface using antibodies after filtration. Antibodies used for flow cytometry were CD45 APC-CY7 (BioLegend, 202216, USA), CD3 APC (BioLegend, 201414, USA), CD4 FITC (BioLegend, 203305, USA), CD45RA PE-CY7 (BioLegend, 202316, USA), CD8 PercP (BioLegend, 201712, USA), CD25 APC (BioLegend, 202114, USA), FoxP3 PE (eBioscience, 17-5773-82, USA). Regulatory T cells (Tregs) were identified using the CD4^+^CD25^+^FoxP3^+^ marker. Briefly, after the addition of respective antibodies, cells were mixed and incubated for 15 min in the dark, and since FoxP3 are expressed in the nucleus, they were treated with fixation/permeabilization (eBioscience, 00-5523-00, USA) for 30 min at 4 °C. All data were analyzed using the FACSCanto II flow cytometer (BD Biosciences, CA, USA) for gating analysis, and all analyses were gated to live cells first.

#### MicroFil vascular perfusion and micro-computed tomography (micro-CT) scanning

Rats were anesthetized and then placed in the supine position with the limbs fixed on a foam board to expose the heart. A perfusion needle was inserted into the left ventricle from the apical site to connect the perfusion pump. Then heparinized saline was perfused into the somatic circulation, followed by 2 min of perfusion using PFA to fix the vessels. Using a 20 ml syringe, the mixed MicroFil compounds were perfused through the perfusion needle into all vessels of the rats. After dehydrating the tissue with gradient alcohol and hyalinizing the tissue with wintergreen oil, the nerves were photographed in a gross view using a stereomicroscope. All nerves were scanned using micro-CT (PerkinElmer, USA) at 70 kV with 114 μA current and 20 μm resolution. Each nerve was scanned for approximately 20 min and the surface area and volume of the vessels were calculated using Analyze 12.0 (AnalyzeDirect, USA).

#### Whole transcriptome RNA sequencing

All experimental procedures were replicated three times to ensure consistency. The technical operations and subsequent analyses were conducted by the Beijing Novozymes Company (Beijing, China). Briefly, RNA was extracted and purified from each sample in each group using Trizol reagent, and agarose gel electrophoresis was then applied to analyze the integrity of RNA and the presence of DNA contamination. Strand-specific libraries were constructed by removing ribosomal RNA (circular RNA (circRNA) library construction and addition of linear RNA removal process), and long noncoding RNAs (lncRNAs), microRNAs (miRNAs), and circRNAs were obtained. Qubit was used for preliminary quantification, and libraries were diluted to 1 ng/µl, and the insert size of the libraries was detected using an Agilent 2100 bioanalyzer. The distribution of the insert size was around 250–300 bp, which was in line with the expectation. The effective concentration of the library was accurately quantified by qPCR to ensure the library quality. In the same principle as mRNA quantification, known lncRNAs and predicted novel_lncRNAs, as well as the known and novel circRNAs in each sample, were identified for expression and normalization.

We adopted whole-transcriptome RNA-sequencing to identify differentially expressed (DE) mRNAs, miRNAs, lncRNAs, and circRNAs. Then Gene Ontology (GO), which includes cellular components (CCs), molecular functions (MFs), and biological processes (BPs), and The Kyoto Encyclopedia of Genes and Genomes (KEGG) enrichment analysis of DE-mRNAs was performed based on the correspondence between lncRNAs and their source genes. The criteria for differential gene screening were padj < 0.05 and | log2(fold change) |> 1.

#### Construction of protein–protein interaction (PPI) network and identification of hub genes

The corresponding PPI networks were constructed and plotted using the DE-mRNA in the STRING system (https://cn.string-db.org/) and Cytoscape software version 3.8.0 (San Diego, CA, USA). These isolated genes that do not interact with other proteins would be removed, and then we get a PPI network map of the target genes. And the top ten hub genes in each group were screened out using the filter parameters of the CytoHubba plugin. In the graph, the colors represent the number of correlations of the expressed modules, with the redder colors indicating more core roles. The pattern of edges denotes the regulatory relationships.

#### Motor function recovery assessment

Paw prints were collected and Gait of walking rats including position, surface area, and pressure of each paw in each group was recorded and analyzed, at 2, 4, 6, 8, 10, and 12 weeks postoperatively using CatWalk XT version 10.6 (Noldus). Based on the paw prints, the system identified the paw print length (PL), toe width (TS), and intermediate toe width (ITS) of the normal side (N) and the injured side (E), and calculated the sciatic function index (SFI), which was calculated as follows: SFI = − 38.3 × (EPL-NPL)/NPL+109.5 × (ETS-NTS)/NTS+13.3 × (EITS-NITS)/NITS-8.8. Generally, the SFI ranges from 0 for healthy nerve function to − 100 for complete dysfunction.

#### Electrophysiological testing

Electrophysiological testing was performed at 12 weeks postoperatively. Briefly, sciatic nerves were exposed bilaterally after the rats were satisfactorily anesthetized, and electrical stimulation (intensity 3 mA) was performed sequentially at the proximal and distal ends of the nerve trunks, with the stimulating electrodes of the Synergy electromyograph (Medtronic Skovlunde) placed at the proximal end of the grafts and the parallel recording electrodes placed at the gastrocnemius muscle of the ipsilateral side, and the compound electromyographic waveforms were recorded in a single stimulation. The normal side was subjected to the same operation. The compound muscle action potential (CMAP) wave amplitude and latency ratio of the injured/normal side muscles for each group were calculated to evaluate the recovery of nerve conduction.

#### Transmission electron microscopy (TEM)

At 12 weeks postoperatively, after the electrophysiological tests, we sampled the nerve tissues 5 mm distal to the grafted segments in each group in order to evaluate the regeneration of nerve fibers and myelin sheaths. Briefly, the obtained neural tissues were immersed in 4% glutaraldehyde solution for 24 h and in 1% osmium tetroxide for 2 h at 4 °C, followed by gradient dehydration in ethanol solution. Ultrathin sections of 70 nm thickness were performed using an ultrathin sectioning machine. Sections were staining using 3% lead citrate-uranyl acetate staining solution, then observed and photographed using a TEM. We counted the myelin thickness in the electron microscopic images of each group and calculated the average myelin thickness. We used Image pro plus 6.0 software to count the myelin thickness in TEM images of each group and calculate the average myelin thickness.

#### Histomorphometric examination of the gastrocnemius muscle

After electrophysiological tests were completed at 12 weeks postoperatively, bilateral gastrocnemius muscles were taken, weighed, and photographed, and the muscle wet-weight ratio was calculated. Subsequently, the muscles of each group were fixed in 4% PFA, dehydrated, embedded, subjected to transverse paraffin sectioning, deparaffinized, and stained using the Masson’s trichrome staining kit, according to the manufacturer’s instructions, and then observed under the microscope and photographed. Then we measured the average cross-sectional area of muscle fibers in each group using Image pro plus 6.0 software.

#### Image analysis

Axonal regeneration lengths (the mean length of NF200-positive staining) were measured using dedicated case viewer software. Subsequently, 3–5 random 40 × microscope fields of view were captured from the fluorescence sections stained for MPZ, CD31, and α-SMA. These fields were used to quantify the extent of regeneration: average fluorescence intensity of regenerated myelin sheath (MPZ-positive staining) and average percentage of the area of functionalized blood vessels (CD31+α-SMA+area/total area) in each field. And the mean thickness of myelin sheath was measured from 3 to 5 areas randomly selected from each TEM image (10x). Image analysis software (Image Pro Plus 6.0) was employed to perform cell counting and quantify early vascular regeneration, axonal outgrowth, and myelin regeneration.

#### Statistical analysis

Both plotting and statistical analysis were performed using GraphPad Prism 8.0 software. Comparisons among three or more groups were performed using one-/two-way ANOVA and Student’s t-test was used for comparison between two groups. All data are expressed as mean ± standard error. Differences between groups were considered statistically significant when *p < 0.05, **p < 0.01, ***p < 0.001, and ****p < 0.0001.

## Results

### Characterization of decellularized nerves

**Fig. 1:**
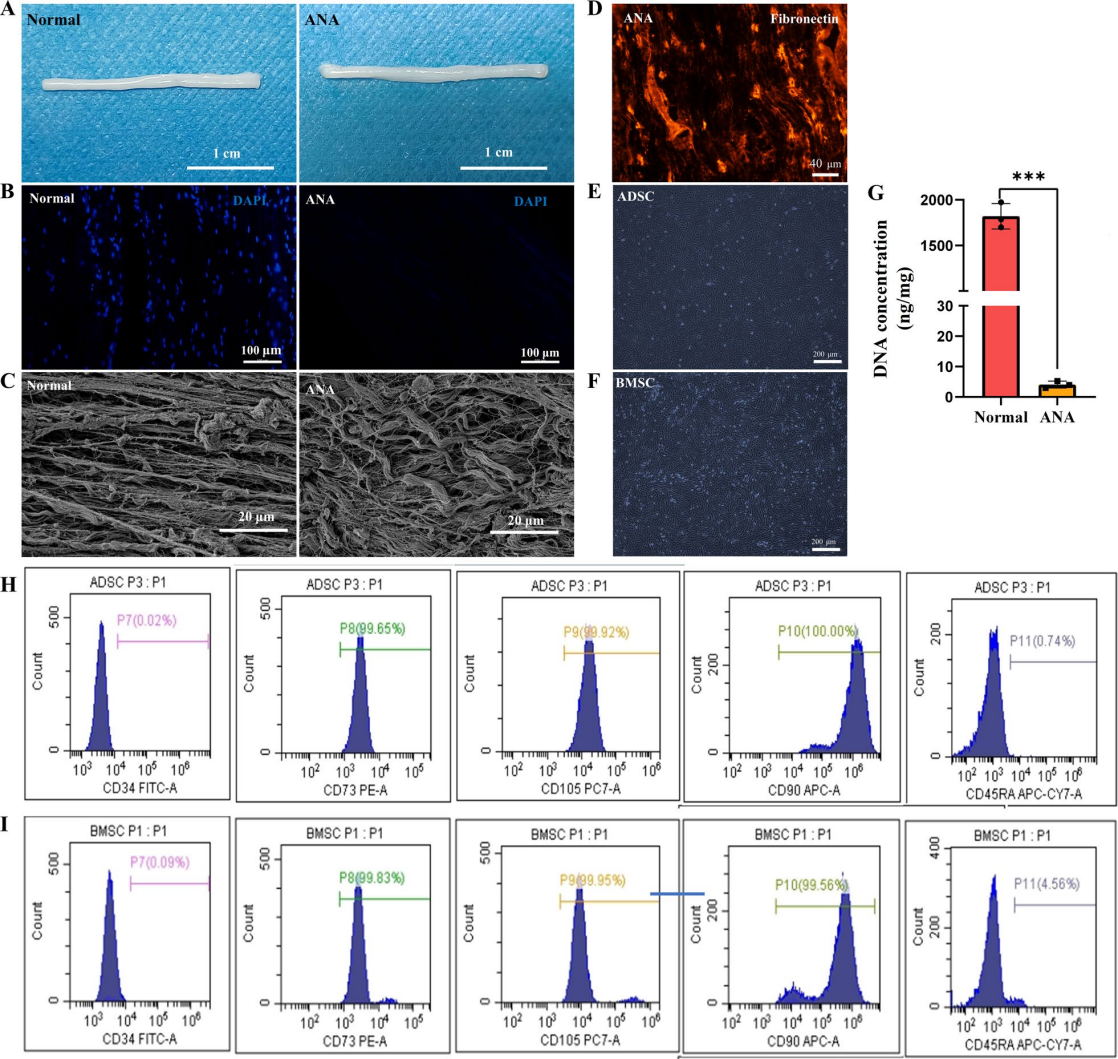
Characterization of decellularized nerve and identification of BMSCs and ADSCs. **A** Gross view of rat sciatic nerve before and after decellularization (scale bar = 1 cm). **B** DAPI staining to observe the nucleus structure of rat sciatic nerve before and after decellularization. **C** Scanning electron micrographs of nerve tissue before and after decellularization, scale bar = 20 μm. **D** Fibronectin staining of decellularized nerves (20x), scale bar = 40 μm. **E**, **F** ADSCs and BMSCs of P3 generation, scale bar = 200 μm. **G** DNA content of normal and decellularized nerves (n = 3). **H**, **I** Flow cytometry analysis of ADSCs and BMSCs. Data represent mean ± standard error and were analyzed using Student’s t test. (***p < 0.001)

After decellularization, the general view of the sciatic nerves showed that decellularized nerves became whiter compared to normal nerves (Fig. 1A). The results of DAPI nuclear staining indicated that most ANA sections were negative, but there existed a negligible DAPI positivity, indicating that the decellularization was basically complete (Fig. 1B). The results of SEM further showed that the decellularized nerve matrix was well-oriented, while the normal nerve still had cellular structures (Fig. 1C). The DNA content test revealed a clear reduction in cellular material after decellularization. Native nerve tissue exhibited a DNA content of 1820.06 ± 80.94, which dropped to 4.09 ± 0.65 following the treatment, indicating effective cell removal (Fig. 1G). In addition, images of tissue sections stained for fibronectin demonstrated that fibronectin, one of the important components of the extracellular matrix, was well preserved after decellularization (Fig. 1D).

### Flow cytometry analysis of ADSCs and BMSCs

Under an inverted phase contrast microscope (IX71, Olympus), the ADSCs and BMSCs of the P3 generation exhibited static adherence and spindle-shaped growth, maintaining a well-grown state (Fig. 1E, F). We performed flow cytometry analysis of extracted primary BMSCs and ADSCs, respectively, and the results showed that ADSCs were strongly positive for MSC surface signature antigens CD73 (99.83%), CD90 (99.56%), and CD105 (99.95%), and were negatively expressed for hematopoietic stem cell surface signature antigens CD34 (0.09%) and CD45 (4.56%) (Fig. 1H). Similarly, BMSCs were positively expressed for MSC surface signature antigens CD73 (99.65%), CD90 (100%), and CD105 (99.92%), with negative expression of hematopoietic stem cell surface signature antigens CD34 (0.02%) and CD45 (0.74%) was observed (Fig. 1I). Collectively, these results indicate that the two types of MSCs were successfully extracted with good stemness.

### Immunomodulatory characteristics of ANA combined with BMSCs or ADSCs in vivo

**Fig. 2:**
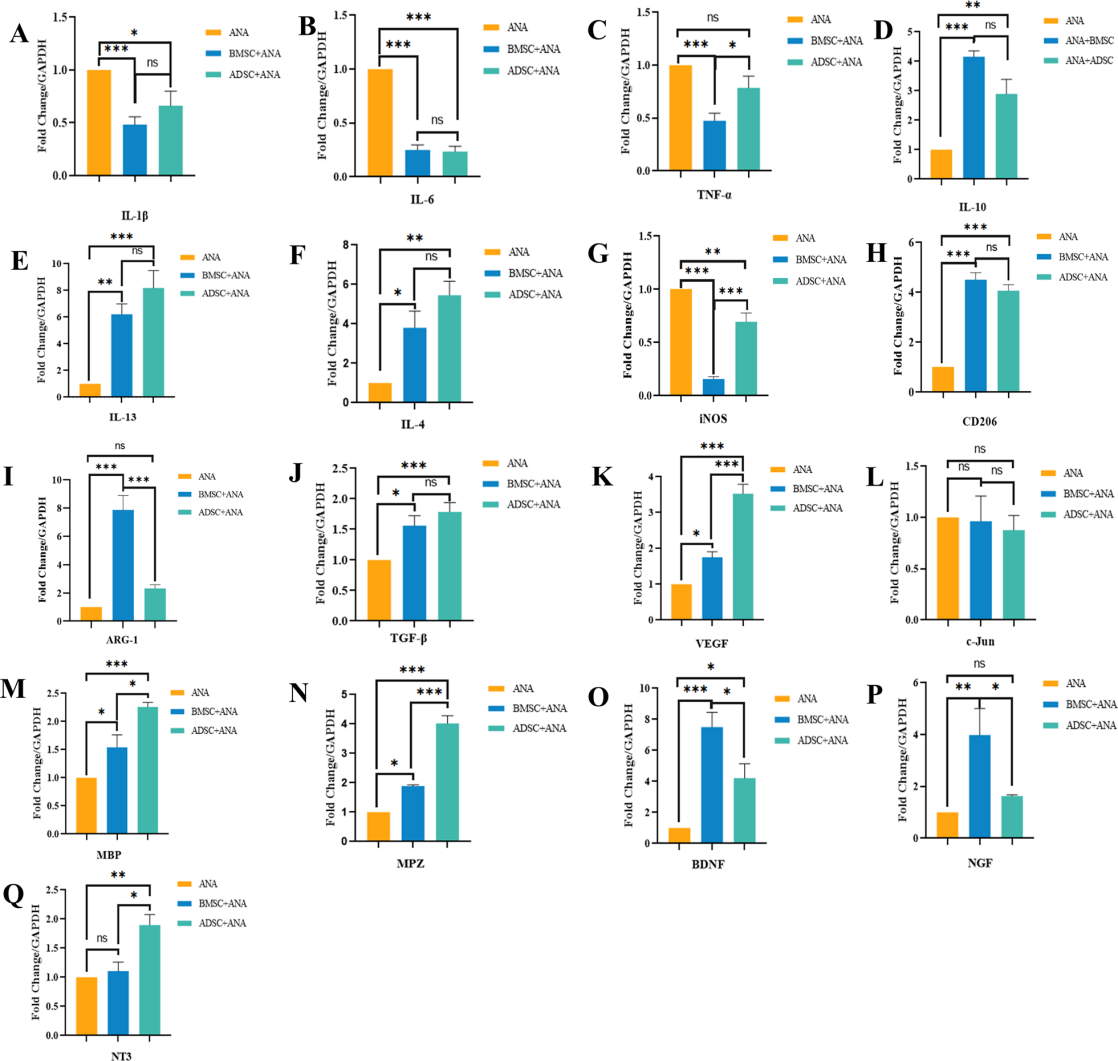
Effects of ANA combined with BMSCs or ADSCs on the gene expression of inflammatory cytokines, myelin-related proteins, pro-angiogenesis-related factors, and neurotrophic factors in each group at 2 weeks postoperatively (n = 5 per group). **A**–**C** Gene expression of pro-inflammatory cytokines IL-1β, IL-6, and TNF-α. **D**–**F** Gene expression of anti-inflammatory cytokines IL-10, IL-13, and IL-4. **G**–**I** Gene expression of M1-type macrophages (iNOS) and M2-type macrophages (CD206, ARG-1). **J**, **K** Gene expression of angiogenesis-related factors TGF-β and VEGF. **L**–**N** Gene expression of myelin production-related proteins c-Jun, MBP, and MPZ. **O**–**Q** Gene expression of neurotrophic factors BDNF, NGF, and NT3. Data represent mean ± standard error and were analyzed using one-way ANOVA. (*p < 0.05, **p < 0.01, ***p < 0.001; ns: no significance)

**Fig. 3:**
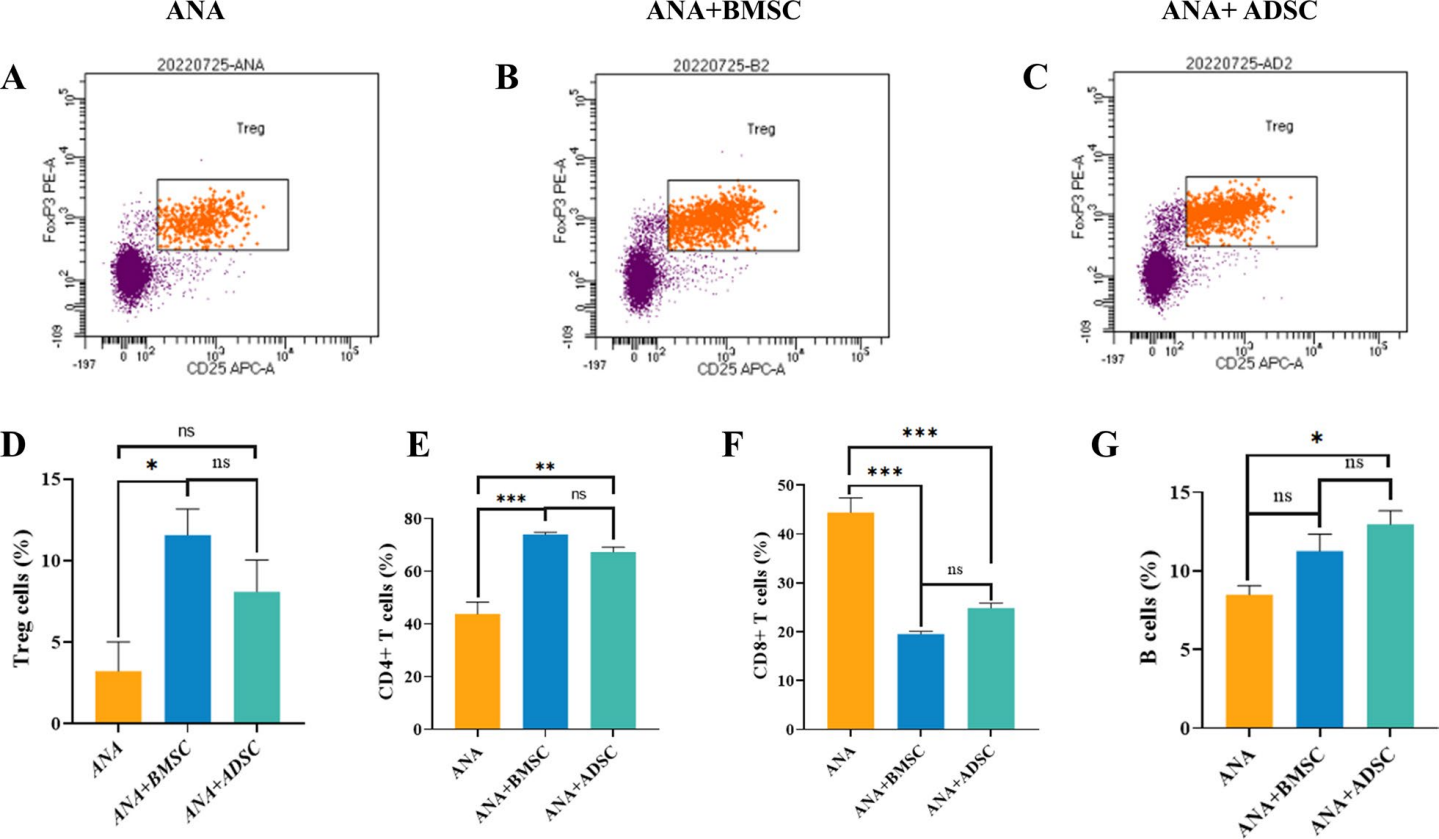
Flow cytometry analysis of the effects of ANA combined with BMSCs or ADSCs on different immune cells at 1 and 2 weeks postoperatively (n = 3). **A**–**C** Flow gating plots of Tregs in each group. **D** Statistical plots of the proportion of Tregs in each group at 2 weeks postoperatively. **E**, **F** Statistical plots of the proportion of CD4+T cells and CD8+T cells in each group at 1 week postoperatively. **G** Statistical graph of the proportion of the number of B cells in each group at 1 week postoperatively. Data represent mean ± standard error and were analyzed using one-way ANOVA. (*p < 0.05, **p < 0.01, ***p < 0.001; ns: no significance)

RT-PCR results showed that the ANA+BMSC group more significantly inhibited the gene expression of pro-inflammatory cytokines IL-1β and TNF-α and promoted the gene expression of anti-inflammatory cytokine IL-10 (Fig. 2A, C, D), as well as efficiently promoted polarization of macrophages from M1-type (iNOS) to M2-type (CD206 and ARG-1) (Fig. 2G–I), while anti-inflammatory cytokines IL-13 and IL-4 were more strongly up-regulated in the ANA+ADSC group (Fig. 2E, F). However, the down-regulation of the pro-inflammatory cytokine IL-6 did not significantly differ between the two groups, and the immunomodulatory effects were stronger in both groups than in the ANA group (Fig. 2B). In addition, the results of flow cytometry analysis showed that ANA+BMSC and ANA+ADSC groups effectively increased the number of B cells and CD4+T cells while decreased the number of CD8+T cells at 1 week postoperatively, in which the effect seemed to be stronger in the ANA+BMSC group, but there was no significant difference between the two groups (Fig. 3E, F, G). Meanwhile, both the ANA+ADSC and ANA+BMSC groups effectively increased the number of Treg cells at 2 weeks postoperatively compared with the ANA group, but the effect of the ANA+BMSC group was more significant than the ANA+ADSC group (Fig. 3A–D). Overall, these data suggested that BMSCs combined with ECM exhibited more significant immunomodulatory effects and helped to maintain immune homeostatic characteristics in vivo.

### ANA combined with BMSCs or ADSCs promotes angiogenesis after PNI

**Fig. 4:**
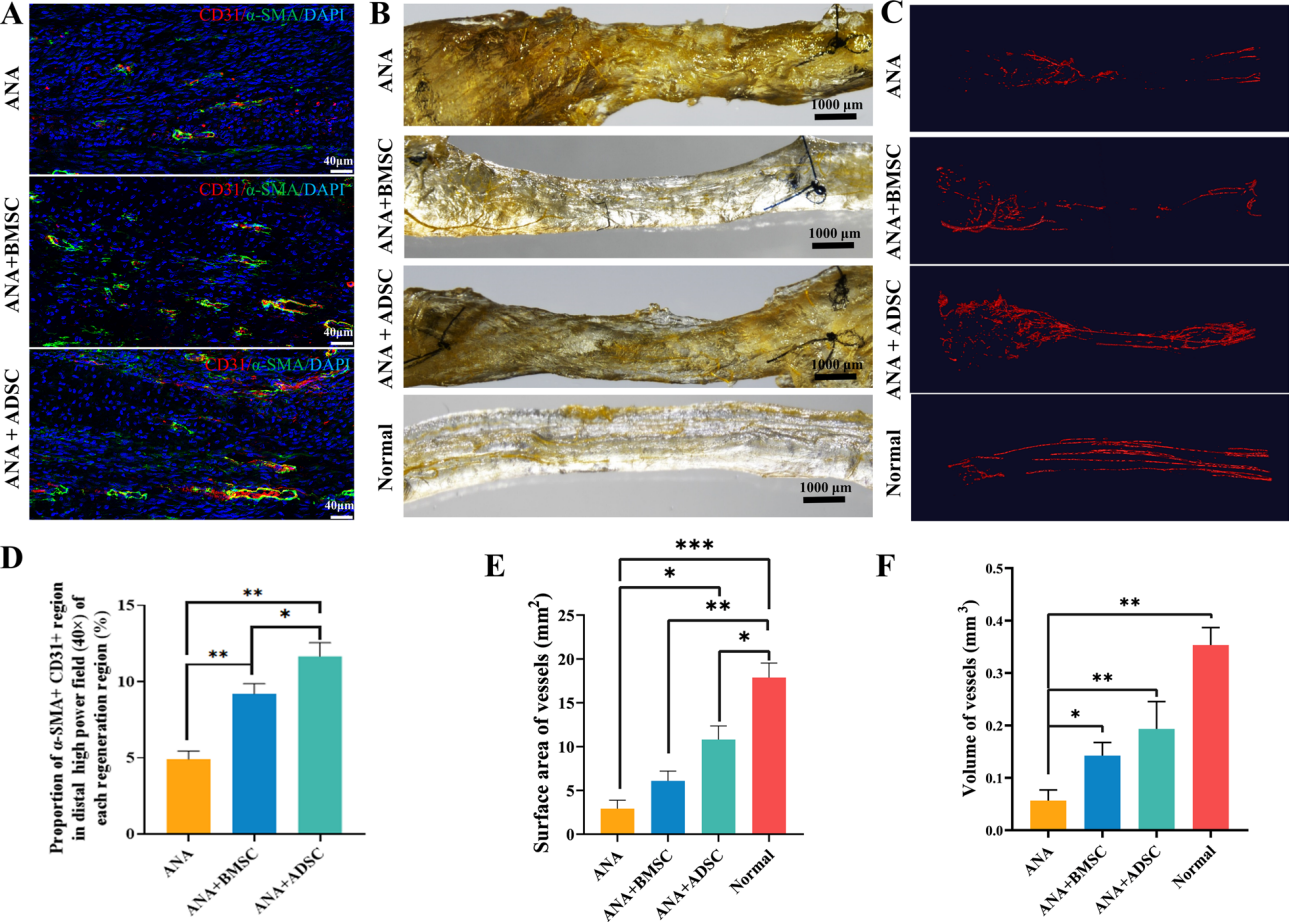
Effects of ANA combined with BMSCs or ADSCs on vascular regeneration at 3 and 4 weeks postoperatively. **A** Immunofluorescence staining of CD31 (endothelial cells, red) and α-SMA (vascular smooth muscle actin, green) in longitudinal sections of neural tissue from each group. (Scale bar = 40 μm). **B** Gross view of neural tissue after MicroFil perfusion of blood vessels in each group at 4 weeks postoperatively, scale bar = 1000 μm. **C** 3D reconstructed image of MicroFil perfused nerve scanned by micro-CT. **D** A statistical plot of the ratio of the area of fluorescent area co-expressed with CD31 and α-SMA to the total area in each group (n = 4 per group). **E**, **F** Total surface area and volume of blood vessels of MicroFil perfused nerve obtained from analysis of micro-CT scans (ANA+BMSC group: n = 3; ANA and ANA+ADSC groups: n = 4). Data represent mean ± standard error and were analyzed using one-way ANOVA. (*p < 0.05, **p < 0.01, ***p < 0.001)

Chronic ischemia, characterized by inadequate blood supply, triggers a cascade of pathological processes in tissues, including oxidative stress, cellular damage, and ultimately, induction of cellular senescence. These processes significantly hinder the regenerative potential of long nerve grafts, where adequate vascularization is crucial for success [31]. To investigate the role of BMSCs and ADSCs on angiogenesis in the presence of tissue-derived ECM materials, we performed RT-PCR for angiogenesis-related factors (TGF-β, VEGF) in the grafts at 2 weeks postoperatively. Significantly, both ANA+ADSC and ANA+BMSC groups increased the gene expression of TGF-β and VEGF compared to those of the ANA group, and the ANA+ADSC group showed significantly higher TGF-β and VEGF levels (Fig. 2J, K). In addition, we performed CD31(endothelial cell) and α-SMA (vascular smooth muscle actin) staining of the grafts in each group at 3 weeks postoperatively and calculated the mean area with double-positive expression in the staining results. The results indicated that the regenerating blood vessels in all three groups were aligned along the direction of nerve regeneration, further suggesting that the nerve-derived ECM exhibited a well-organized natural orientation structure that can support oriented regeneration of axons and blood vessels (Fig. 4A). Especially, ANA with ADSCs significantly promoted the growth of regenerating blood vessels, which was superior to the ANA+BMSC group by calculating the average regenerated area of blood vessels in each group (Fig. 4D). Additionally, we performed MicroFil angiography of the grafts in each group at 4 weeks postoperatively to obtain a gross view of the perfused grafts (Fig. 4B), followed by micro-CT scanning to determine the surface area and volume of vessels within the grafts. The results indicated that the normal nerve blood supply was continuous with the largest vascular surface area and volume. The ANA+ADSC group exhibited a significantly greater pro-vascular regeneration effect compared to the ANA+BMSC group. Notably, both groups demonstrated enhanced vascularization compared to the ANA group alone (Fig. 4C, E, F). Taken together, these data indicated that ANA with ADSCs exhibited more significant promotion of vascular regeneration characteristics after PNI.

### ANA combined with BMSCs or ADSCs up-regulates the expression of neurotrophic factors

Neurotrophic factors, such as BDNF, NGF, and NT3, are known to regulate the local microenvironment of nerve regeneration and promote axon growth [32, 33]. RT-PCR results showed that ANA+BMSC and ANA+ADSC groups significantly up-regulated the expression of BDNF, NGF, and NT-3 compared with the ANA group, with the ANA+BMSC group more significantly up-regulating BDNF and NGF (Fig. 2O, P), while the ANA+ADSC group more significantly up-regulating NT-3 (Fig. 2Q).

### ANA combined with BMSCs or ADSCs promotes myelin regeneration

**Fig. 5:**
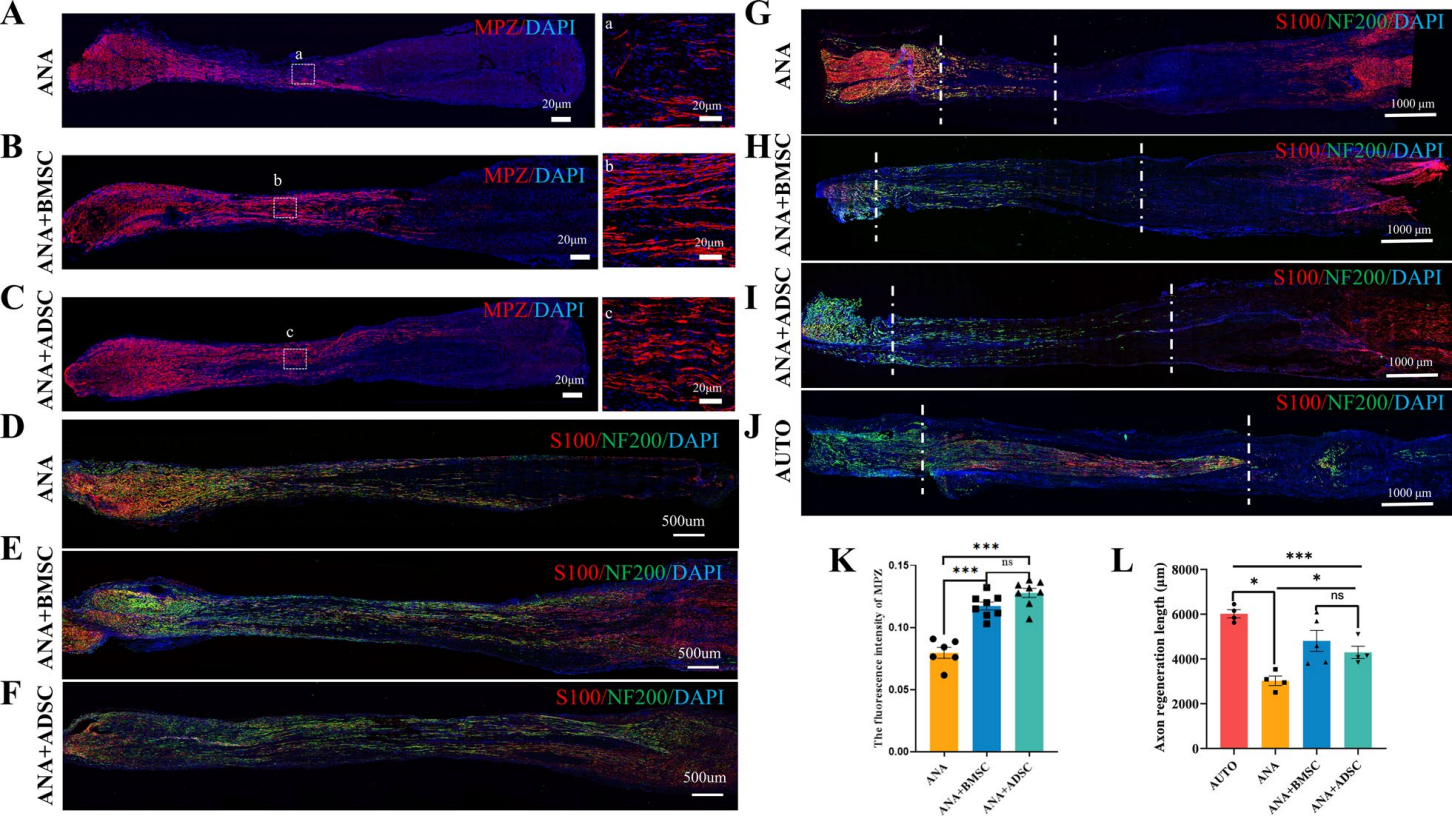
Effects of ANA combined with BMSCs or ADSCs on axonal regeneration and intraneural myelin regeneration at 2 and 3 weeks postoperatively. **A**–**C** Immunofluorescence staining of myelin-associated protein MPZ in ANA, ANA+BMSC, and ANA+ADSC groups at 3 weeks postoperatively. **A**–**C** represents the respective local magnification. Scale bar = 20 μm. **D**–**F** Immunofluorescence staining results of axons (NF200) and Schwann cells (S100) in each group at 3 weeks postoperatively. Scale bar = 500 μm. **G**–**J** Immunofluorescence staining results of axons (NF200) and Schwann cells (S100) in each group at 2 weeks postoperatively. Scale bar = 1000 μm. **K** Statistical graphs of the fluorescence intensity of MPZ at 3 weeks postoperatively. **L** Statistical graph of axon regeneration length in each group at 2 weeks postoperatively (AUTO, ANA+ADSC and ANA groups: n = 4; ANA+BMSC group: n = 5). Data represent mean ± standard error and were analyzed using one-way ANOVA. (*p < 0.05, ***p < 0.001; ns: no significance)

RT-PCR results revealed that compared with the ANA group, ANA+ADSC and ANA+BMSC groups effectively promoted the expression of mature-type myelin-associated protein genes (MPZ, MBP), with the ANA+ADSC group exhibiting stronger up-regulation (Fig. 2M, N), but no significant effect was observed in the immature-type myelin gene (c-Jun) (Fig. 2L). To further verify their pro-myelinogenic effects, we performed immunofluorescence staining of MPZ (a primary myelin-related protein) in the grafts at 3 weeks postoperatively (Fig. 5A–C), and the results showed that the statistically superior length of myelin extension and fluorescence intensity of MPZ were better in the ANA+ADSC group than in the ANA+BMSC group, both of which were superior to the ANA group (Fig. 5K, L). Taken together, these data indicated that both BMSCs and ADSCs effectively promoted myelin production under the effect of tissue-derived ECM, with ADSCs showing more significant pro-myelin production characteristics.

### DE-miRNAs during ANA combined with BMSCs or ADSCs repair of PNI

**Fig. 6:**
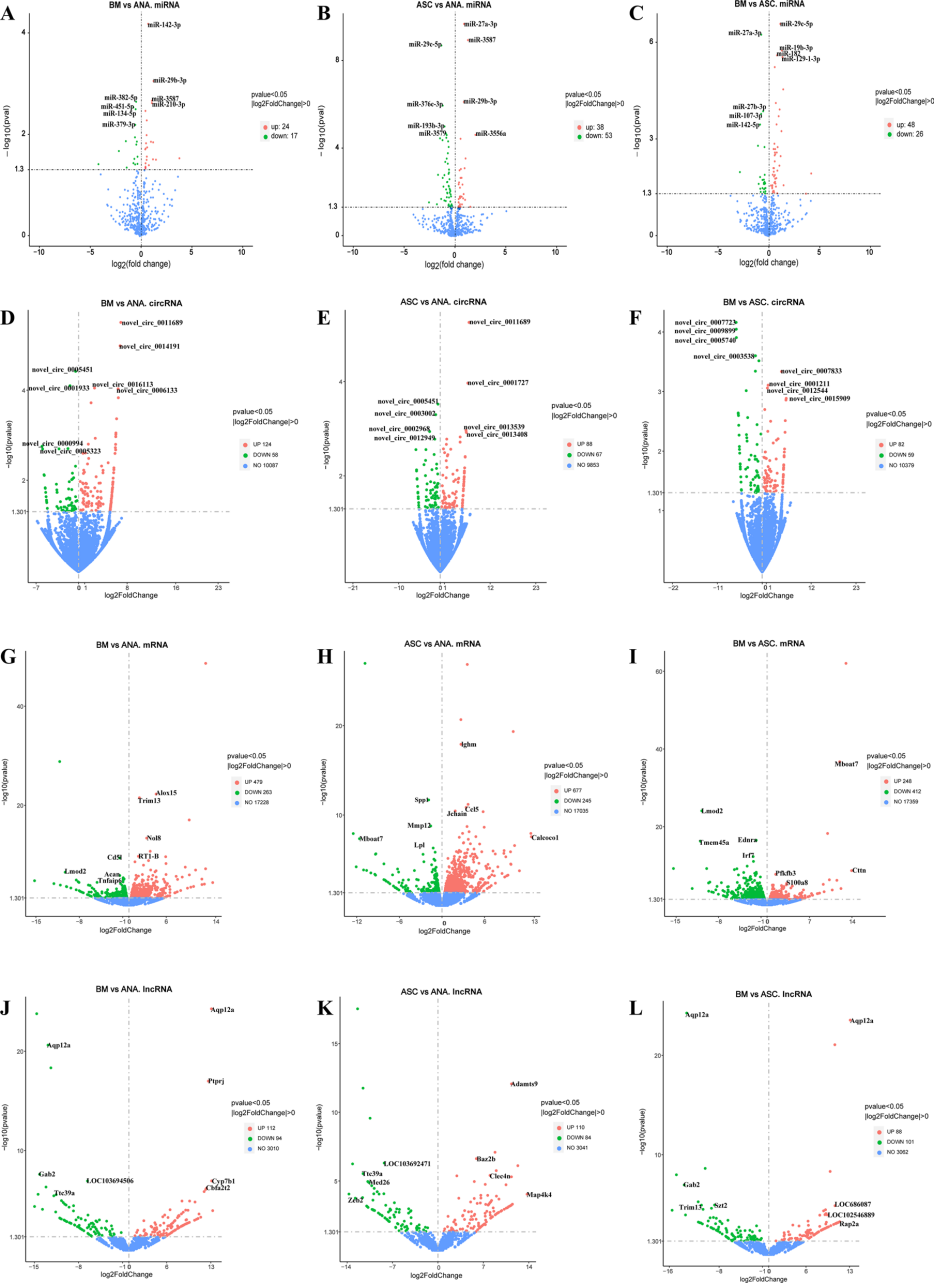
Expression profiles of distinct RNAs at 2 weeks postoperatively (n = 9). In the volcano plots, red, green, and blue colors represent up-regulated differential RNAs, down-regulated differential RNAs, and RNAs with no significant difference. **A**–**C** DE-miRNAs of BM vs ANA, ASC vs ANA, and BM vs ASC. **D**–**F** DE-circRNAs of BM vs ANA, ASC vs ANA, and BM vs ASC. **G**–**I** DE-circRNAs in the ANA+BMSC group compared with the ANA group and the ANA+ADSC group. **G**–**I** DE-circRNAs in the BMSC group compared with the ANA group and the ANA+ADSC group. **G**–**I** DEmRNAs of BM vs ANA, ASC vs ANA, and BM vs ASC. **J**–**L** DE-lncRNAs of BM vs ANA, ASC vs ANA, and BM vs ASC. x-axis: log2 ratio of miRNAs to miRNAs. axis: log2 ratio of miRNA/circRNA/mRNA/lncRNA expression levels. y-axis: false discovery rate values (− log10 transformed) of miRNA/circRNA/mRNA/lncRNA. BM: ANA+BMSC group; ASC: ANA+ADSC group; ANA: ANA group. DE: differentially expressed

From the above experimental results, it was revealed that ADSCs- or BMSCs combined with ANA differ in inflammatory regulation, vascularization, and myelin generation in vivo, respectively. Then we used whole transcriptome sequencing to explore the potential molecular mechanisms of ANA combined with BMSCs or ADSCs during repairing PNI. The volcano plot results showed that 24 DE-miRNA expressions were up-regulated and 17 DE-miRNA expressions were down-regulated in the ANA+BMSC group compared with the ANA group, respectively (Fig. 6A). miR-142-3p—which is an inflammation-associated miRNA—negatively regulates pro-inflammatory mediators, including nuclear factor-kappa B (NF-κB), TNF-α, and IL-6 [34]. Elevated miR-29b expression was found to inhibit neuronal apoptosis induced by spinal cord injury and ethanol neurotoxicity [35]. While human umbilical cord-derived mesenchymal stem cell extracellular vesicles (hUC-MSCs-EVs) can promote neurological recovery in spinal cord injury rats by up-regulating miR-29b-3p [36], inhibition of miR-29b-3p expression enhances the expression of microglial pro-inflammatory factors such as iNOS [37]. miR-210-3p is associated with the regulation of human umbilical vein endothelial cell (HUVEC) angiogenesis, and its inhibition affects the proliferation, migration, survival, and angiogenesis of HUVECs [38]. Thirty-eight DE-miRNAs were up-regulated and 53 DE-miRNAs were down-regulated in the ANA+ADSC group (Fig. 6B). Among them, elevated miR-27a-3p has been shown to have neuroprotective effects, and it also promotes proliferation and migration and inhibits the apoptotic ability of human cevocytes [39, 40]. miR-3587 has only been found to inhibit iron death in renal tubular epithelial cells [41]. A total of 48 DE-miRNAs were up-regulated and 26 DE-miRNAs were down-regulated in the ANA+BMSC group compared with the ANA+ADSC group (Fig. 6C). Among them, miR-182 was shown to inhibit endothelial cell apoptosis [42].

### DE-circRNAs during ANA combined with BMSCs or ADSCs repair of PNI

The results of the DE-circRNA volcano plot showed that 124 DE-circRNAs were up-regulated and 58 DE-circRNAs were down-regulated in the ANA+BMSC group compared with the ANA group (Fig. 6D). In total, eighty-eight DE-circRNAs were up-regulated and 67 DE-circRNAs were down-regulated in the ANA+ADSC group compared with the ANA group (Fig. 6E). Overall, 82 DE-circRNAs were up-regulated and 59 DE-circRNAs were down-regulated in the ANA+BMSC group compared with the ANA+ADSC group (Fig. 6F). CircRNAs can inhibit the miRNA function by binding to miRNAs [43]. Therefore, miRNA binding site analysis of the identified circRNAs can help to further investigate the function of circRNAs.

### DE-mRNAs and -lncRNAs during ANA combined with BMSCs or ADSCs repair of PNI

The volcano plot results revealed that the ANA+BMSC group had 479 up-regulated DE-mRNAs and 263 down-regulated DE-mRNAs compared with the ANA group. After removing genes whose names are not yet known, we obtained the significantly up-regulated genes Alox15, Trim13, Nol8 and RT1-B, and the significantly down-regulated genes Cd5l, Lmod2, Acan, and Tnfaip6 (Fig. 6G). Cd5l, a soluble protein of the scavenger receptor cysteine-rich (SRCR) superfamily that is expressed mainly in macrophages and lymphocytes in inflamed tissues, is a key protein involved in the regulation of immune homeostasis and inflammatory diseases, and can also regulate leukocyte migration, signal transduction, and apoptotic and metabolic processes [44]. The ANA+ADSC group had 677 up-regulated DE-mRNAs and 245 down-regulated DE-mRNAs compared with the ANA group. Among them, the significantly up-regulated genes were Ighm, Ccl5, Jchain and Calcoco1, and the significantly down-regulated genes were Spp1, Mmp12, Mboat7 and Lpl (Fig. 6H). The ANA+BMSC group had 248 up-regulated DE-mRNAs and 412 down-regulated DE-mRNAs compared with the ANA+ADSC group. Among them, we also obtained the significantly up-regulated genes Mboat7, Cttn, Pfkfb3 and S100a8, and the significantly down-regulated genes Lmod2, Ednra, Tmem45a, Irf7 and Mx1 (Fig. 6I). Cttn, which is upstream of the PI3K-AKT signaling pathway, inhibits apoptosis [45, 46]**.**

**Fig. 7:**
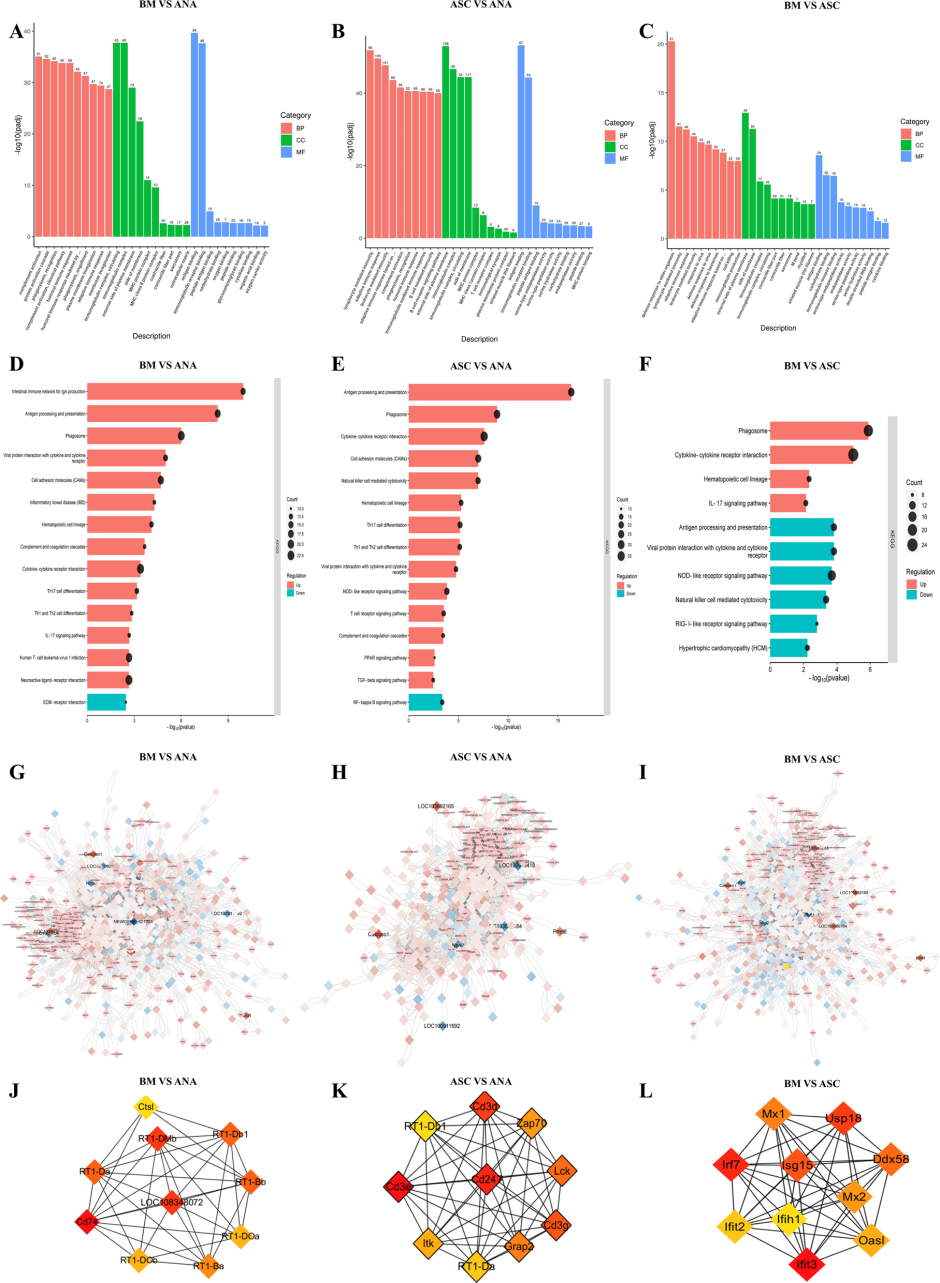
lncRNA-mRNA transcriptomic analysis of BM vs ANA, ASC vs ANA, and BM vs ASC at 2 weeks postoperatively. The top 30 most significantly enriched GO terms of differential RNAs. Different colors represent the three GO subclasses biological processes (BPs, pink), cellular components (CCs, green), and molecular functions (MFs, blue). **A** GO enrichment analysis of DE-mRNAs in the ANA+BMSC group compared with the ANA group. **B** GO enrichment analysis of DE-mRNAs in the ANA+ADSC group compared with the ANA group. **C** GO enrichment analysis of DE-mRNAs in the ANA+BMSC group compared with the ANA+ADSC group. **D** Line plots of the significantly enriched KEGG pathways of DE-mRNAs in the ANA+BMSC group compared with the ANA group. **E** Line plots of the significantly enriched KEGG pathways of DE-mRNAs in the ANA+ADSC group compared with the ANA group. **F** Line plots of the significantly enriched KEGG pathways of the DE-mRNAs in the ANA+BMSC group compared with the ANA+ADSC group. Bars indicate the size of the *p*-value, the longer the bar, the smaller the *p*-value; dots indicate the number of genes, and the larger the dot, the greater the number of genes. **G** PPI network of DE-mRNAs in the ANA+BMSC group compared with the ANA group **H** PPI network of DE-mRNAs in the ANA+ADSC group compared with the ANA group. **I** PPI network of DE-mRNAs in the ANA+BMSC group compared to the ANA+ADSC group. **J** Top 10 hub genes in the PPI network of the DE-mRNAs in the ANA+BMSC group compared with the ANA group. **K** Top 10 hub genes in the PPI network of the DE-mRNAs in the ANA+ADSC group compared with the ANA group. **L** Top 10 hub genes in the PPI network of the DE-mRNAs in the ANA+BMSC group compared to the ANA+ADSC group. Darker colors represent higher degree in the network. DE: differentially expressed

The biological functions of lncRNAs include regulating the expression of target genes at both transcriptional and post-transcriptional levels and identifying the differential functional pathways generated by different treatments [47]. The results of the volcano plot showed that 112 DE-lncRNAs were up-regulated and 94 DE-lncRNAs were down-regulated in the ANA+BMSC group compared with the ANA group (Fig. 6J). The ANA+ADSC group had 110 up-regulated DE-lncRNAs and 84 down-regulated DE-lncRNAs compared with the ANA group (Fig. 6K). A total of 88 DE-lncRNAs were up-regulated and 101 DE-lncRNAs were down-regulated in the ANA+BMSC group compared with the ANA+ADSC group (Fig. 6L). All differential genes are summarized in Supplementary Tables 1–3. Since the sample size was less than 5, we only chose to predict the target genes of lncRNAs by their location regarding protein-coding genes (Co-location, i.e., analyzed by looking for genes within 100 kb upstream and downstream of the lncRNA) [48]. The results of GO enrichment analysis of differentially expressed genes (DEGs) revealed that BPs such as complement activation, protein activation cascade, phagocytosis, and recognition, CCs such as immunoglobulin complex, circulating immunoglobulin complex, and outer plasma membrane, and MFs such as antigen-binding and immunoglobulin receptor-binding, coagulation factor receptor-binding, and growth factor-binding were highly enriched in the ANA+BMSC group as compared with the ANA group (Fig. 7A). The result of KEGG pathway enrichment analysis showed the DEGs were mainly enriched in pathways such as the Intestinal immune network for IgA production, cytokine-cytokine receptor interaction, Cell adhesion molecules (CAMs), among others (Fig. 7D). The DEGs of the ANA+ADSC group were mainly enriched in lymphocyte-mediated immunity, adaptive immune response, leukocyte-mediated immunity, and other biological processes; external side of the plasma membrane, immunoglobulin complex and other cellular components; and antigen binding, immunoglobulin receptor binding, and peptide antigen binding, and other molecular functions (Fig. 7B). Furthermore, KEGG pathway enrichment analysis indicated that DEGs were mainly enriched in cytokine-cytokine receptor interaction, CAMs, natural killer (NK) cell-mediated cytotoxicity, and other processes (Fig. 7E). Cell-to-cell interactions are mediated by a large number of cell adhesion molecules and often involve cytoskeletal rearrangements [49, 50]. DEGs were mainly enriched in adaptive immune response, leukocyte-mediated immunity, and lymphocyte-mediated immunity in the ANA+BMSC group compared with the ANA+ADSC group (Fig. 7C). Moreover, the results of KEGG pathway enrichment analysis demonstrated that DEGs were mainly enriched in Phagosome, IL-17 signaling, cytokine-cytokine receptor interaction, and NOD-like receptor signaling pathways (Fig. 7F).

### Construction of DE-mRNAs mediating PPI network

We constructed a PPI network for DE-mRNAs with a significance threshold of *p* value < 0.05 using the STRING system. Subsequently, we visualized the networks for BM vs. ANA, ASC vs. ANA, and BM vs. ASC groups in Cytoscape (Fig. 7G–I). To identify key genes within each group, we applied filter parameters from the cytoHubba plug-ins. The node color reflects the degree of hub gene importance, with redder nodes indicating higher degrees. The top key genes identified in the BM vs. ANA group include Cd74, RT1-Da, RT1-DMb, and RT1-Bb (Fig. 7J). The top key genes identified in the ASC vs. ANA group were Cd247, Cd3e, Cd3d, Cd3g, Lck, and Grap2 (Fig. 7K). And the top key genes identified in the BM vs. ASC group included Irf7, Ifit3, Usp18, Isg15, Ddx58 (Fig. 7L). Cd3e and Cd3d were involved in pathways such as hematopoietic cell lineage Th17 cell differentiation, human T-cell leukemia virus 1 infection, Th1 and Th2 cell differentiation, human immunodeficiency virus 1 infection, and T cell receptor signaling. Cd247 was involved in pathways such as NK cell-mediated cytotoxicity, Th17 cell differentiation, Th1 and Th2 cell differentiation, T cell receptor signaling, etc. Cd3g was involved in pathways such as Hematopoietic cell lineage, human T-cell leukemia virus 1 infection, Th17 cell differentiation, Th1 and Th2 cell differentiation, and T cell receptor signaling. Lck was involved in pathways such as NK cell-mediated cytotoxicity, Th17 cell differentiation, Th1 and Th2 cell differentiation, T cell receptor signaling, and NF-κB signaling. In the ANA+BMSC group, Cd74 was involved in Antigen processing and presentation. RT1-Da, RT1-DMb, and RT1-Bb were involved in the Intestinal immune network for IgA production, Antigen processing and presentation, Phagosome, CAMs, Hematopoietic cell lineage, Th17 cell differentiation, Th1 and Th2 cell differentiation, etc. Irf7 was involved in NOD-like receptor signaling and RIG-I-like receptor signaling pathways. Usp18 and Isg15 were involved in the RIG-I-like receptor signaling pathway. Th17 cells, which have a wide range of functions in immune regulation and disease, can be divided into pathogenic and non-pathogenic Th17 cells [51], both of which can differentiate in response to different cytokine stimuli; for instance, IL-6, IL-23, and IL-1β drive the differentiation of pathogenic Th17 cells, which can express numerous pro-inflammatory cytokines, such as granulocyte–macrophage colony-stimulating factor (GM-CSF) and IL-23R and a few anti-inflammatory factors, including IL-10 and CD5L. T cells can express numerous pro-inflammatory cytokines, such as GM-CSF and IL-23R, and a few anti-inflammatory factors, such as IL-10 and CD5L. TGF-β and IL-6 drive non-pathogenic Th17 cell differentiation, which may express small amounts of GM-CSF and IL-23R and numerous IL-10 and CD5L to promote immune homeostasis [52–54]. NOD-like receptors are key regulators of immune responses and are also involved in tumorigenesis and angiogenesis [55]. The RIG-I-like receptor signaling pathway can regulate innate and adaptive immune responses by acting on interferon-alpha (IFN-α) and IFN-β and pro-inflammatory cytokines and influencing chemokine production [56].

### Evaluation of axon regeneration and motor functional recovery

Given the variability in the characteristics of the two MSCs combined with ANA at the molecular level, we further compared the effectiveness of them in repairing PNI. To further investigate the effects of BMSCs or ADSCs combined with ANA on axon regeneration after PNI, we performed S100 and NF200 staining of the nerve grafts of each group, at 2 and 3 weeks postoperatively, respectively (Fig. 5G–J). By measuring the mean length of axon growth in each group at 2 weeks postoperatively, we found that the AUTO group had the best repair effect, while the mean length of axonal growth in the ANA+BMSC group was slightly longer than that in the ANA+ADSC group, but there was no significant difference, and both were better than that in the ANA group (Fig. 5M). Moreover, the NF200 and S100 staining results at 3 weeks postoperatively showed that the axons in the ANA+BMSC and the ANA+ADSC groups had already grown into the distal end, which was better than that in the ANA group (Fig. 5D–F).

**Fig. 8:**
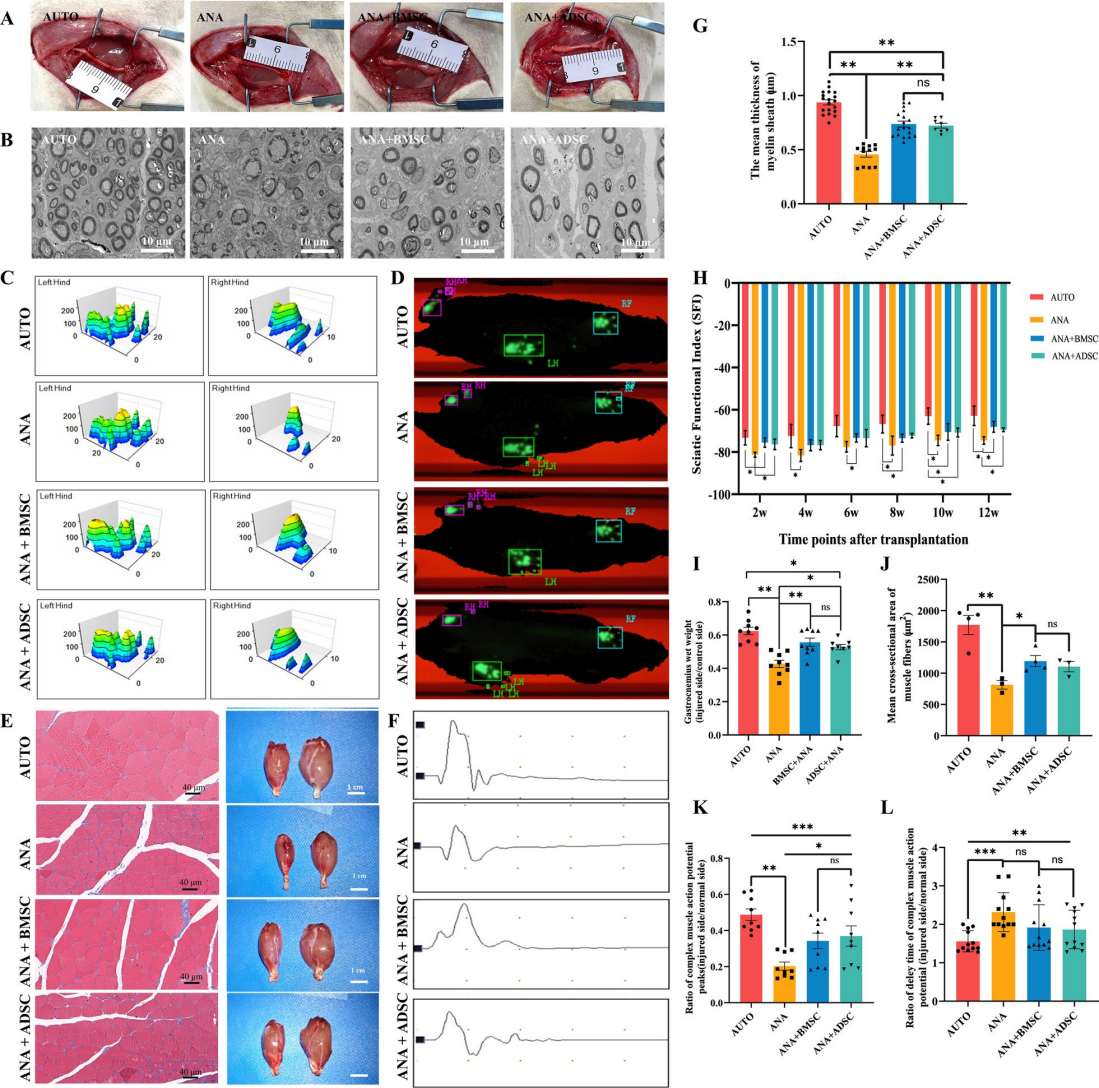
ANA combined with BMSCs or ADSCs promoted peripheral nerve regeneration and motor function recovery at 12 weeks postoperatively. **A** Gross view of nerve grafts of each group at 12 weeks postoperatively. **B** The representative TEM images of each group at 12 weeks postoperatively. Scale bar = 10 μm. **C** The three-dimensional (3D) footprint intensities charts of CatWalk gait analysis. And the intensities range from 0 to 255. **D** 2D paw print maps in Catwalk gait for each group. **E** Masson staining of cross sections of the gastrocnemius muscle on the injured side in each group. Scale bar = 40 μm. And the gross view of the gastrocnemius muscle of the injured (left) and normal (right) sides of each group. Scale bar = 1 cm. **F** Compound muscle action potential (CMAPs) waveforms on the injured side of each group at 12 weeks postoperatively. **G** Quantification of the mean thickness of myelin sheath of injured side in each group (n = 3 per group). **H** Sciatic nerve function index (SFI) of each group at 2, 4, 6, 8, 10, and 12 weeks postoperatively. **I** The ratio of gastrocnemius muscle wet weight (injured side/normal side) in each group (AUTO, ANA and ANA+BMSC groups: n = 9; ANA+ADSC: n = 8). **J** The mean cross-sectional area of gastrocnemius muscle fibers in each group at 12 weeks postoperatively. **K** Ratio of complex muscle action potentials peak in each group (injured side/normal side) (AUTO and ANA+BMSC groups: n = 4; ANA and ANA+ADSC groups: n = 3). **L** Ratio of delay time of complex muscle action potential in each group (injured side/normal side). Data represent mean ± standard error and were analyzed using one-way ANOVA (**G**, **I**, **J**, **K**, **L**) and two-way ANOVA (**H**). (*p < 0.05, **p < 0.01, ***p < 0.001; ns: no significance)

At 12 weeks postoperatively, from the general view of the nerve grafts at the time of sampling, we found that the nerve grafts in the AUTO group grew close to normal nerves, whereas the nerve grafts in the ANA group were thinned (Fig. 8A). Furthermore, representative TEM images illustrating regenerating nerves distal to nerve grafts in each group illustrate the myelin sheath regeneration results. Notably, the autograft group exhibited a compact and regularly rounded profile of the myelin sheath. In the ANA+ADSC and ANA+BMSC groups, while not as compact as the AUTO group, the regenerating myelin sheaths displayed a regular and subcircular profile similar to the autograft group. In contrast, TEM images of the ANA group revealed sparse and fragmented myelin sheaths (Fig. 8B). The mean thickness analysis of myelin sheaths further confirmed these observations, showing no significant difference between the ANA+ADSC and ANA+BMSC groups (Fig. 8G).

### BMSCs or ADSCs combined with ANA promote sciatic nerve function recovery

At 12 weeks postoperatively, gait analysis was performed using Catwalk XT system every two weeks starting from 2 weeks postoperatively to assess the recovery of target muscle function. The 2D paw print showed that in the AUTO, ANA+BMSC and ANA+ADSC group, all the clarity and completeness of the injured side were better than in the ANA group (Fig. 8D). And the 3D paw prints demonstrated the contact area and average strength of each paw print with the plate (Fig. 8C). Both 2D and 3D visualization of paw prints revealed a uniform distribution of plantar force on the healthy side, while the injured side exhibited varying degrees of altered plantar force. Moreover, SFI results indicated a decrease in sciatic function across all groups from 2 to 4 weeks postoperatively, followed by a gradual increase from 6 to 12 weeks postoperatively. The AUTO group exhibited the most substantial recovery of sciatic nerve function compared to other groups. Both the ANA+ADSC and ANA+BMSC groups demonstrated effective promotion of functional recovery after PNI, with no significant difference between the two groups, and both outperformed the ANA group (Fig. 8H). Data obtained from photographing and weighing the gastrocnemius muscle, calculating the muscle wet weight recovery rate, and performing Masson staining to determine the muscle cross-sectional area revealed that there was no significant difference between the ANA+BMSC group and the ANA+ADSC group (Fig. 8E, I, J). We further performed electrophysiological evaluation and found that the CMAP wave amplitude and latency ratio (injured side/control side) were significantly lower in the ANA group than in ANA+ADSC and ANA+BMSC groups, with no significant difference between the latter two groups (Fig. 8F, K, L). Collectively, these data suggest that although BMSCs and ADSCs exhibit different characteristics during early repair of PNI in the ECM, they have similar pro-axonal regeneration and functional restoration effects during the long-term repair of PNI in rats.

## Discussion

MSC therapy, which is known to promote intraneural angiogenesis and facilitate neuronal proliferation and survival by inhibiting inflammatory responses and pro-apoptotic pathways, is currently a very promising strategy for the clinical treatment of PNI [26, 27, 57]. Nerve tissue-derived ECM has also been shown to regulate functions such as adhesion and survival of SCs, promote myelin formation and axon growth, and affect cell behaviors such as cell growth, proliferation, migration, and differentiation [58, 59]. Zhou et al. [60] found that BMSCs and ADSCs have similar pro-proliferative effects on SCs in vitro. Moreover, when ADSCs and BMSCs were used to bridge sciatic nerve defects in rats after injection into decellularized nerve grafts, respectively, they were found to have similar pro-neural regenerative and functional repair functions at 16 weeks postoperatively. On this basis, our study sought to investigate the in vivo biological properties of two types of MSCs combined with tissue-derived ECM and the potential molecular mechanisms underlying their pro-neural repair, as well as further characterising the DEGs of the two types of MSCs combined with ANA using whole transcriptomics approaches to better assist clinical decision-making in selecting the most suitable MSCs for the treatment of PNI. In our study, we firstly found that ANA combined with BMSCs or ADSCs exerted different repair characteristics. On the one hand, ANA combined with BMSCs more significantly modulated the immune microenvironment, such as more significantly up-regulating gene expression of IL-10 and down-regulating gene expression of IL-1β and TNF-α, promoting macrophage polarization from M1 to M2 type, and increasing the number of Tregs than ANA combined with ADSCs in vivo. On the other hand, ANA combined with ADSCs is more characterized in promoting angiogenesis and myelination, such as more significantly up-regulating the gene expression of angiogenesis-related factors VEGF and TGF-β, and myelin-associated protein MBP and MPZ than ANA combined with BMSCs. Additionally, the results of α-SMA and CD31staining, as well as those of MicroFil vascular perfusion tests, further indicated that ANA combined with ADSCs had a more significant effect on the promotion of early angiogenesis after PNI.

Immune cells, especially M2 macrophages and Tregs, and inflammatory cytokines play an essential role in repairing PNI. For instance, IL-10, one of the most potent endogenous counter-regulators of proinflammatory factors, exhibits neuroprotective properties and plays an important role in inhibiting neuropathic pain [61, 62]. Moreover, IL-10 may regulate macrophage recruitment, fostering neurorestorative effects within the initial 3 days following PNI, and it also participate in inflammation regulation and contributes to the promotion of peripheral nerve regeneration, impacting the recovery of motor and sensory functions [63]. And it has been found that M2 macrophages exert anti-inflammatory and pro-neural regenerative effects, contributing to alleviate local inflammation and provide trophic factors, whereas IL-10 also helps to modulate the immune response and induces macrophage polarization to the M2-type [64, 65]. In addition, Tregs also play an important role in maintaining microenvironmental immune homeostasis by suppressing the inflammatory activity of innate and adaptive immune cells and secreting pro-tissue repair cytokines to counteract inflammation [66], such as the absorption of IL-2, a key cytokine needed to eliminate inflammatory cell survival. Moreover, CD4, CD35, and Tregs exert neuroprotective properties and promote neuronal survival [67, 68]. Studies have shown that BMSCs inhibit the differentiation of CD4+T cells to Th17 cells [69, 70]. BMSCs were found to play an important role in regulating CD4+T cell-mediated adaptive immune cell responses by secreting IL-6, hepatocyte growth factor (HGF), prostaglandin F2 (PGF2), iNOS and TGF-β1 [71]. However, we didn’t find there was significant difference between ANA combined BMSCs and ANA combined ADSCs in regulating CD4+T cells.

Notably, regeneration of blood vessel and vascular network reconstruction are very essential for peripheral nerve regeneration [72]. In this process, VEGF is a key regulator of angiogenesis, vascular remodeling, and vascular permeability and is involved in inducing inflammation and mucin accumulation, contributing to wound healing [73–75]. Previous studies comparing the function of ADSCs and BMSCs in secreting VEGF and protein expression in vitro found no significant difference between them, and repairing diabetic wounds after combining the two MSCs with collagen revealed no significant difference in the number of α-SMA- and CD31-positive cells between the two, as well as a similar cell survival and migration function within the scaffold after transplantation [76]. However, we can’t definitively assert that VEGF is directly secreted by our transplanted ADSCs in vivo. It is plausible that various cell types, including endothelial cells, might have been recruited, contributing to the elevated expression of cytokine genes in the transplanted segments. This aspect warrants further in-depth investigation. In addition, among the top 10 hub genes of the ANA+ADSC group compared to the ANA group, we found that they appear to be associated with angiogenesis, axonal growth, and myelin formation. For instance, Lck is a key regulator of T cell activation and differentiation [77] and it also serves as a mediator of β-integrin signal transduction to regulate the migration of SCs along the axon and cytoskeletal rearrangement, axon sorting, and myelin formation. It was found that mice with Lck gene knockout exhibited delayed myelin formation, abnormal myelin thickness, and dysfunctional radial sorting of axons [78]. Ddx58, also known as retinoic acid-inducible gene-1 (RIG-1), was found to play an important role in angiogenesis, endothelial cell hyperpermeability, and NO production, as well as exerts antiviral activity during viral infection [75, 79]. And endothelin receptor type A (Ednra), which was significantly down-regulated genes in the ANA+BMSC group compared with the ANA+ADSC group, is highly expressed by vascular wall cells, including smooth muscle cells and pericytes, and is also present in human vascular endothelial cells. It can mediate vasoconstrictive growth and inflammation occurrence [80, 81]. Therefore, we hypothesize that the pro-regenerative effects of ANA combined with ADSCs are more through the regulation of vascularization and myelin regeneration, as well as the upregulation of cytokine expression such as VEGF and NT-3, which may involve the regulation of key genes such as Lck, Ednra, Ddx58 and so on.

In addition, it has been found that ADSCs or BMSCs injected into the ANA and transplanted in vivo for repairing peripheral nerve defect are still viable around 28 days postoperatively [82, 83]. Notably, it can be inferred that the final survival time of MSCs after injection into the ANA is unclear, and whether this also affects the long-term repair outcome need to be clarified. Lastly, our findings allow us to identify the specific repair characteristics exhibited in the early stages of nerve repair by the two types of MSCs combined with nerve tissue-derived ECM material, along with the associated molecular mechanisms, thus both achieved good pro-peripheral nerve regeneration results. We speculate that this is the result of the interaction between the neural tissue-derived extracellular matrix and MSCs. However, because peripheral nerve regeneration involves a very complex regenerative microenvironment, we cannot conclusively determine whether the changes in cytokine genes are directly secreted by the MSCs themselves or if they enhance the secretory function of the cells entering the grafted segments, which needs further in-depth study.

## Conclusion

Overall, the current study demonstrated that ANA combined with ADSCs is characterized by enhanced pro-angiogenic and myelinogenic properties, whereas ANA with BMSCs exhibits a more prominent immunomodulatory profile. The molecular mechanisms underlying these distinctions influence the process of nerve regeneration. Despite not demonstrating significant differences in pro-regenerative and functional recovery effects during the long-term repair of PNI, aligning with previous studies. However, there are some limitations to our study, for instance, we did not further validate the differential genes and key pathways and explore the final survival time of ADSCs and BMSCs in vivo, as well as we could not conclude how much the ECM actually plays a role in this different repair characteristics exhibited by ADSCs and BMSCs. In addition, whether a moderate increase in the number of MSCs transplanted in vivo could further enhance the pro-neural repair effect was not verified in our study. Currently, our focus has predominantly been on the role of DE-mRNAs, with insufficient attention given to DE-circRNAs, DE-lncRNAs, and DE-miRNAs. This warrants further in-depth exploration, especially as the clinical application of MSCs becomes more widespread.

### Supplementary Information

Additional file 1. 

Additional file 2. 

Additional file 3.

## Data Availability

All the datasets of RNA-seq included in this study have been uploaded to the Gene Expression Omnibus (GEO) database (GSE266596) and are publicly accessible at https://www.ncbi.nlm.nih.gov/geo/query/acc.cgi?acc=GSE266596 after the release date of Jun 08, 2024. The authors confirm the availability of supporting data within this article and its supplemental information.

## Abbreviations

ECM: Extracellular matrix
MSC: Mesenchymal stem cell
ADSCs: Adipose-derived mesenchymal stem cells
BMSCs: Bone marrow-derived mesenchymal stem cells
ANA: Acellular nerve allograft
IL: Interleukin
TNF-α: Tumor necrosis factor-alpha
Tregs: Regulatory T cells
lncRNAs: Long noncoding RNAs
circRNAs: Circular RNAs
miRNAs: MicroRNAs
mRNAs: Messenger RNAs
PNI: Peripheral nerve injury
SCs: Schwann cells
PDGF: Platelet-derived growth factor
EGFR: Epidermal growth factor receptor
VEGFR: Vascular endothelial growth factor receptor
GDNF: Glial cell line-derived neurotrophic factor
NGF: Nerve growth factor
BDNF: Brain-derived neurotrophic factor
SD: Sprague–Dawley
ddH_2_O: Double-distilled water
PBS: Phosphate-buffered saline
SDS: Sodium dodecyl sulfate
SEM: Scanning electron microscopy
DMEM: Dulbecco’s Modified Eagle Medium
FITC: Fluorescein isothiocyanate
PE: Phycoerythrin
HLA: Human leukocyte antigen
APC: Allophycocyanin
PFA: Paraformaldehyde
DAPI: 4’,6-diamidino-2-phenylindole
NF200: Neurofilament 200
MPZ: Myelin protein zero
iNOS: Inducible nitric oxide synthase
ARG-1: Arginase-1
RT-PCR: Real-time quantitative polymerase chain reaction
CT: Computed tomography
DE: Differentially expressed
GO: Gene ontology
CCs: Cellular components
MFs: Molecular functions
BPs: Biological processes
KEGG: Kyoto Encyclopedia of Genes and Genomes
PPI: Protein–protein interaction
PL: Print length
TS: Toe width
ITS: Intermediate toe width
SFI: Sciatic function index
CMAP: Compound muscle action potential
TEM: Transmission electron microscopy
GM-CSF: Granulocyte–macrophage-colony-stimulating factor
HGF: Hepatocyte growth factor
PGF2: Prostaglandin F2
NT-3: Neurotrophin-3
SRCR: Scavenger receptor cysteine-rich
DEGs: Differentially expressed genes
CAMs: Cell adhesion molecules
NK: Natural killer
IFN-α: Interferon-alpha
Lck: Lymphocyte-specific protein tyrosine kinase
RIG-1: Retinoic acid-inducible gene-1
Ednra: Endothelin receptor type A
NF-κB: Nuclear factor-keppaB
hUC-MSCs-EVs: Human umbilical cord-derived mesenchymal stem cell extracellular vesicles
HUVEC: Human umbilical vein endothelial cell
