# Supplementary material for: Biological characteristics of tissue engineered-nerve grafts enhancing peripheral nerve regeneration
Source: Stem Cell Res Ther. 2024 Jul 18;15:215. doi: 10.1186/s13287-024-03827-9 (PMC11256578; doi:10.1186/s13287-024-03827-9)
Supplement: Supplementary file 1 — Additional file 1. [file 13287_2024_3827_MOESM1_ESM.docx]

**Supplementary Table 1 Differentially expressed genes of lncRNA (BM vs ANA)**

| Gene ID | Gene name | Log2(Fold Change) | padj | Type |
| --- | --- | --- | --- | --- |
| ENSRNOG00000004452 | Aqp12a | 13.09972 | 4.19E-21 | lncRNA |
| XLOC_012648 | XLOC_012648 | -14.6301 | 1.05E-20 | lncRNA |
| ENSRNOG00000004452 | Aqp12a | -12.8343 | 1.10E-17 | lncRNA |
| XLOC_027339 | XLOC_027339 | -12.3811 | 1.68E-15 | lncRNA |
| ENSRNOG00000034025 | Ptprj | 12.60772 | 2.33E-14 | lncRNA |
| ENSRNOG00000011882 | Gab2 | -14.1745 | 1.87E-05 | lncRNA |
| ENSRNOG00000009730 | Cyp7b1 | 13.12767 | 7.01E-05 | lncRNA |
| ENSRNOG00000046062 | LOC103694506 | -6.57647 | 7.01E-05 | lncRNA |
| XLOC_016985 | XLOC_016985 | -13.1553 | 0.000234 | lncRNA |
| ENSRNOG00000016352 | Cbfa2t2 | 12.31228 | 0.000296 | lncRNA |
| XLOC_004169 | XLOC_004169 | 12.20068 | 0.000368 | lncRNA |
| ENSRNOG00000029001 | RT1-S2 | 11.94647 | 0.000549 | lncRNA |
| XLOC_012648 | XLOC_012648 | -14.4135 | 0.000963 | lncRNA |
| ENSRNOG00000047573 | Ttc39a | -11.542 | 0.001023 | lncRNA |
| ENSRNOG00000060518 | AABR07015057.1 | -11.9179 | 0.001289 | lncRNA |
| ENSRNOG00000012270 | Med26 | -10.7022 | 0.003402 | lncRNA |
| ENSRNOG00000057054 | LOC103690118 | 8.42853 | 0.003601 | lncRNA |
| XLOC_018506 | XLOC_018506 | 9.640708 | 0.003601 | lncRNA |
| ENSRNOG00000023257 | Adamts9 | 10.49356 | 0.004436 | lncRNA |
| XLOC_028844 | XLOC_028844 | -10.4141 | 0.00487 | lncRNA |
| XLOC_001428 | XLOC_001428 | -10.1823 | 0.006483 | lncRNA |
| ENSRNOG00000002443 | Mfap3 | -9.96358 | 0.007382 | lncRNA |
| ENSRNOG00000028659 | Szt2 | -8.7833 | 0.008287 | lncRNA |
| ENSRNOG00000047746 | AABR07000398.1 | -14.9722 | 0.009383 | lncRNA |
| ENSRNOG00000009075 | Trim13 | -9.49562 | 0.010453 | lncRNA |
| XLOC_021500 | XLOC_021500 | -13.717 | 0.014726 | lncRNA |
| ENSRNOG00000053790 | AABR07036007.1 | 6.541145 | 0.015561 | lncRNA |
| XLOC_001428 | XLOC_001428 | 9.020504 | 0.01652 | lncRNA |
| XLOC_015314 | XLOC_015314 | 9.387877 | 0.016886 | lncRNA |
| ENSRNOG00000014013 | Map4k4 | 13.30291 | 0.017768 | lncRNA |
| XLOC_009888 | XLOC_009888 | -8.8009 | 0.018639 | lncRNA |
| ENSRNOG00000034139 | Lyc2 | -11.6198 | 0.022666 | lncRNA |
| ENSRNOG00000045574 | Morn3 | 8.6293 | 0.023022 | lncRNA |
| XLOC_015314 | XLOC_015314 | 8.341443 | 0.031079 | lncRNA |
| XLOC_001428 | XLOC_001428 | 11.12785 | 0.031306 | lncRNA |
| ENSRNOG00000019352 | Emc6 | 5.687659 | 0.03638 | lncRNA |
| ENSRNOG00000010489 | Samd4a | -6.49587 | 0.03638 | lncRNA |
| ENSRNOG00000057994 | AABR07034718.2 | 7.952393 | 0.039223 | lncRNA |
| ENSRNOG00000053033 | AABR07061022.2 | 7.850983 | 0.042271 | lncRNA |
| ENSRNOG00000061006 | AABR07017208.2 | 7.964703 | 0.043203 | lncRNA |
| ENSRNOG00000049908 | AABR07037436.1 | 5.380626 | 0.047372 | lncRNA |
| ENSRNOG00000059679 | LOC103692165 | 12.37228 | 5.18E-45 | mRNA |
| ENSRNOG00000014685 | LOC108348167 | -11.1627 | 1.73E-25 | mRNA |
| ENSRNOG00000019183 | Alox15 | 4.389817 | 4.08E-19 | mRNA |
| ENSRNOG00000009075 | Trim13 | 1.695824 | 1.66E-18 | mRNA |
| ENSRNOG00000046246 | AABR07015881.1 | 9.723302 | 4.16E-14 | mRNA |
| ENSRNOG00000046968 | Nol8 | 2.863976 | 1.37E-10 | mRNA |
| ENSRNOG00000048070 | AABR07051563.1 | 3.430716 | 5.44E-08 | mRNA |
| ENSRNOG00000032708 | RT1-Bb | 1.527351 | 5.05E-07 | mRNA |
| ENSRNOG00000030332 | AABR07065670.1 | 5.926235 | 5.53E-07 | mRNA |
| ENSRNOG00000023068 | Cd5l | -1.51586 | 8.82E-07 | mRNA |
| ENSRNOG00000020951 | Slc4a1 | 3.159111 | 2.61E-06 | mRNA |
| ENSRNOG00000004402 | Lpgat1 | 0.971617 | 4.17E-06 | mRNA |
| ENSRNOG00000059199 | AABR07065705.4 | 5.977283 | 8.81E-06 | mRNA |
| ENSRNOG00000049829 | AABR07060872.1 | 2.350975 | 1.75E-05 | mRNA |
| ENSRNOG00000048458 | AABR07065705.1 | 9.086153 | 3.86E-05 | mRNA |
| ENSRNOG00000059447 | AC109901.2 | 12.35715 | 7.49E-05 | mRNA |
| ENSRNOG00000050000 | AABR07034739.1 | 3.510937 | 0.000123 | mRNA |
| ENSRNOG00000050994 | Cttn | 10.74447 | 0.000123 | mRNA |
| ENSRNOG00000053527 | AABR07034736.1 | 4.38259 | 0.000164 | mRNA |
| ENSRNOG00000056052 | AABR07060980.1 | 4.205775 | 0.000259 | mRNA |
| ENSRNOG00000045831 | Lmod2 | -10.2775 | 0.000284 | mRNA |
| ENSRNOG00000049243 | AABR07065656.3 | 5.983212 | 0.000718 | mRNA |
| ENSRNOG00000046834 | C3 | 2.171995 | 0.000718 | mRNA |
| ENSRNOG00000047790 | B9d1 | 9.743774 | 0.000829 | mRNA |
| ENSRNOG00000058426 | AABR07065656.9 | 3.857418 | 0.000961 | mRNA |
| ENSRNOG00000029980 | Zbtb16 | 1.151122 | 0.000961 | mRNA |
| ENSRNOG00000028992 | Acan | -1.4755 | 0.001013 | mRNA |
| ENSRNOG00000060572 | Tlk1 | 1.538534 | 0.001246 | mRNA |
| ENSRNOG00000029001 | RT1-S2 | 7.617412 | 0.001342 | mRNA |
| ENSRNOG00000054828 | AABR07051708.1 | 3.178584 | 0.00154 | mRNA |
| ENSRNOG00000056285 | AABR07065693.3 | 4.397276 | 0.001778 | mRNA |
| ENSRNOG00000054513 | LOC103693776 | 8.592847 | 0.001778 | mRNA |
| ENSRNOG00000050792 | Tnfaip6 | -0.9204 | 0.002309 | mRNA |
| ENSRNOG00000010478 | Serpina3n | 1.225416 | 0.00255 | mRNA |
| ENSRNOG00000039754 | Rab7b | -0.76635 | 0.003534 | mRNA |
| ENSRNOG00000012181 | Lpl | -0.94322 | 0.003551 | mRNA |
| ENSRNOG00000047415 | AABR07060788.1 | 11.76116 | 0.00359 | mRNA |
| ENSRNOG00000050102 | AABR07065699.1 | 3.697414 | 0.003618 | mRNA |
| ENSRNOG00000052069 | AABR07065673.1 | 5.587708 | 0.003618 | mRNA |
| ENSRNOG00000047599 | AABR07065802.1 | 7.846694 | 0.003802 | mRNA |
| XLOC_007243 | XLOC_007243 | 1.051053 | 0.00383 | mRNA |
| ENSRNOG00000010663 | Col6a5 | 3.068354 | 0.004164 | mRNA |
| ENSRNOG00000034190 | Ighm | 2.167988 | 0.004408 | mRNA |
| ENSRNOG00000003734 | AABR07041600.1 | -8.49263 | 0.004698 | mRNA |
| ENSRNOG00000043451 | Spp1 | -0.91905 | 0.005355 | mRNA |
| ENSRNOG00000022009 | Mzb1 | 2.342199 | 0.005676 | mRNA |
| ENSRNOG00000052925 | NEWGENE_621351 | -15.213 | 0.006262 | mRNA |
| ENSRNOG00000053103 | AABR07065705.2 | 4.36143 | 0.006839 | mRNA |
| ENSRNOG00000056828 | AABR07065705.3 | 5.760603 | 0.008727 | mRNA |
| ENSRNOG00000018711 | Ppcdc | -8.18854 | 0.009292 | mRNA |
| ENSRNOG00000047103 | AABR07065656.1 | 4.57188 | 0.009292 | mRNA |
| ENSRNOG00000048402 | AABR07065625.2 | 2.125564 | 0.009292 | mRNA |
| ENSRNOG00000030187 | Mmp12 | -0.94989 | 0.009772 | mRNA |
| ENSRNOG00000033017 | LOC100910669 | -5.13841 | 0.011486 | mRNA |
| ENSRNOG00000055719 | AABR07065656.7 | 5.829143 | 0.011486 | mRNA |
| ENSRNOG00000020109 | LOC100910371 | -8.04565 | 0.011486 | mRNA |
| ENSRNOG00000049814 | LOC100910882 | 13.50486 | 0.012054 | mRNA |
| ENSRNOG00000047571 | RGD1563231 | 4.106139 | 0.013691 | mRNA |
| ENSRNOG00000059121 | AABR07065714.1 | 3.938733 | 0.013942 | mRNA |
| ENSRNOG00000046452 | Fcgr2b | 0.890006 | 0.016282 | mRNA |
| ENSRNOG00000036877 | Clca4l | 6.214452 | 0.017449 | mRNA |
| ENSRNOG00000050275 | LOC100912399 | -12.0974 | 0.018083 | mRNA |
| ENSRNOG00000010507 | Mmp7 | -1.35207 | 0.018123 | mRNA |
| ENSRNOG00000058824 | AABR07065789.3 | 3.069838 | 0.018255 | mRNA |
| ENSRNOG00000018148 | LOC108348081 | 2.710204 | 0.020836 | mRNA |
| ENSRNOG00000050864 | Cpne1 | 12.16737 | 0.020836 | mRNA |
| ENSRNOG00000054391 | Snurf | 1.693495 | 0.021562 | mRNA |
| ENSRNOG00000014378 | Il1r2 | 2.201598 | 0.024104 | mRNA |
| ENSRNOG00000048982 | Calcoco1 | 11.77462 | 0.024582 | mRNA |
| ENSRNOG00000008602 | Steap4 | 0.727111 | 0.026202 | mRNA |
| ENSRNOG00000031848 | AABR07065651.1 | 3.97607 | 0.029212 | mRNA |
| ENSRNOG00000008210 | Ky | -6.53564 | 0.029212 | mRNA |
| ENSRNOG00000004247 | Nhp2 | -11.0551 | 0.029212 | mRNA |
| ENSRNOG00000003666 | Jchain | 1.683996 | 0.030356 | mRNA |
| ENSRNOG00000010253 | Cd163 | 1.283335 | 0.030659 | mRNA |
| ENSRNOG00000060917 | AABR07050298.1 | 6.049787 | 0.030851 | mRNA |
| ENSRNOG00000046393 | LOC100360647 | 1.418579 | 0.030851 | mRNA |
| ENSRNOG00000049766 | Sctr | -1.10381 | 0.03135 | mRNA |
| ENSRNOG00000047365 | LOC687780 | 1.388129 | 0.03135 | mRNA |
| ENSRNOG00000058590 | AABR07034739.2 | 1.929107 | 0.031354 | mRNA |
| ENSRNOG00000028896 | A2m | 0.878265 | 0.03276 | mRNA |
| ENSRNOG00000053228 | AABR07061036.1 | -6.2553 | 0.03276 | mRNA |
| ENSRNOG00000048062 | AABR07065815.1 | 2.736511 | 0.032836 | mRNA |
| ENSRNOG00000011500 | Pou2af1 | 1.75474 | 0.033061 | mRNA |
| ENSRNOG00000022523 | Fkbp5 | 0.775161 | 0.034274 | mRNA |
| ENSRNOG00000052050 | AABR07065651.7 | 4.196765 | 0.035145 | mRNA |
| ENSRNOG00000004854 | Has2 | -0.68893 | 0.038906 | mRNA |
| ENSRNOG00000017597 | Fbp1 | 3.087844 | 0.038947 | mRNA |
| ENSRNOG00000051768 | AABR07065693.2 | 3.801408 | 0.038947 | mRNA |
| ENSRNOG00000030235 | AABR07065823.2 | 3.369409 | 0.04093 | mRNA |
| ENSRNOG00000036703 | Itgax | -0.79132 | 0.045004 | mRNA |
| ENSRNOG00000045786 | AABR07065693.1 | 6.546803 | 0.045164 | mRNA |
| ENSRNOG00000047658 | AABR07034718.1 | 10.42263 | 0.045164 | mRNA |
| ENSRNOG00000055700 | AC096473.3 | 0.76744 | 0.045164 | mRNA |
| ENSRNOG00000048935 | Tmem45a | -10.0774 | 0.045793 | mRNA |
| ENSRNOG00000045677 | Nlrp6 | -10.0271 | 0.050067 | mRNA |
| ENSRNOG00000042083 | AABR07051562.1 | 2.3687 | 0.054226 | mRNA |
| ENSRNOG00000002934 | Atp1b1 | -0.65787 | 0.055969 | mRNA |
| ENSRNOG00000046854 | AABR07061022.1 | 9.982397 | 0.058977 | mRNA |
| ENSRNOG00000017198 | Hif3a | 4.565759 | 0.061057 | mRNA |
| ENSRNOG00000009465 | Sfrp2 | -0.81734 | 0.066095 | mRNA |
| ENSRNOG00000014532 | Lbp | 0.708987 | 0.067197 | mRNA |
| ENSRNOG00000008478 | Mmp13 | 1.089878 | 0.070825 | mRNA |
| ENSRNOG00000050859 | AABR07065772.1 | 4.646995 | 0.070825 | mRNA |
| ENSRNOG00000001823 | St6gal1 | 1.057323 | 0.072445 | mRNA |
| ENSRNOG00000015668 | Ccl19 | 5.925823 | 0.074522 | mRNA |
| ENSRNOG00000026898 | Zbtb8b | -5.93995 | 0.074522 | mRNA |
| ENSRNOG00000052047 | AABR07051718.1 | 3.531494 | 0.076608 | mRNA |
| ENSRNOG00000002314 | LOC103689943 | 0.767146 | 0.077694 | mRNA |
| ENSRNOG00000001742 | LOC108348101 | -9.36353 | 0.077813 | mRNA |
| ENSRNOG00000020982 | Fau | -1.01557 | 0.07952 | mRNA |
| ENSRNOG00000046572 | AABR07065651.2 | 3.255481 | 0.07952 | mRNA |
| ENSRNOG00000017187 | Prmt6 | 9.445798 | 0.080534 | mRNA |
| ENSRNOG00000050509 | Ebi3 | 1.319968 | 0.08065 | mRNA |
| ENSRNOG00000006736 | Ccr3 | 1.472397 | 0.08079 | mRNA |
| ENSRNOG00000049315 | AABR07065827.1 | 2.443511 | 0.08079 | mRNA |
| ENSRNOG00000054620 | AC135026.1 | 3.710615 | 0.081262 | mRNA |
| ENSRNOG00000059322 | N4bp2l1 | 1.49999 | 0.082118 | mRNA |
| ENSRNOG00000029776 | AABR07065823.1 | 4.212772 | 0.082878 | mRNA |
| ENSRNOG00000020836 | Rorc | 1.82408 | 0.085906 | mRNA |
| ENSRNOG00000002947 | Dpt | -0.58084 | 0.085906 | mRNA |
| ENSRNOG00000057165 | AABR07034730.3 | 3.115969 | 0.087215 | mRNA |
| ENSRNOG00000015336 | Isl2 | 5.657629 | 0.087504 | mRNA |
| ENSRNOG00000007457 | Serping1 | 0.605322 | 0.098512 | mRNA |
| ENSRNOG00000003543 | Gpr143 | -2.00165 | 0.09942 | mRNA |
| ENSRNOG00000010181 | Clec4d | 0.824276 | 0.100773 | mRNA |
| ENSRNOG00000033215 | RT1-Db1 | 0.910311 | 0.101284 | mRNA |
| ENSRNOG00000056495 | AABR07065676.1 | 5.3752 | 0.104189 | mRNA |
| ENSRNOG00000022711 | Slco4c1 | 2.721682 | 0.104282 | mRNA |
| ENSRNOG00000018238 | Nuggc | 3.248926 | 0.107392 | mRNA |
| ENSRNOG00000037418 | Inca1 | -5.65101 | 0.115053 | mRNA |
| ENSRNOG00000015903 | Add2 | 2.074782 | 0.123421 | mRNA |
| ENSRNOG00000032844 | RT1-Da | 0.819284 | 0.123608 | mRNA |
| ENSRNOG00000049331 | LOC685048 | 6.626357 | 0.129616 | mRNA |
| ENSRNOG00000011557 | S100a8 | 1.833993 | 0.133321 | mRNA |
| ENSRNOG00000047005 | Kcnk5 | 2.50382 | 0.133812 | mRNA |
| ENSRNOG00000006738 | Fbxo32 | 0.691477 | 0.133812 | mRNA |
| ENSRNOG00000049755 | AABR07051534.1 | 5.151146 | 0.133812 | mRNA |
| ENSRNOG00000014137 | Fbln1 | 0.588755 | 0.134029 | mRNA |
| ENSRNOG00000055428 | AABR07060963.1 | 3.718054 | 0.136306 | mRNA |
| ENSRNOG00000007091 | Ly6e | -1.00322 | 0.136306 | mRNA |
| ENSRNOG00000002593 | Esrrg | -3.44032 | 0.136306 | mRNA |
| ENSRNOG00000014956 | Slc11a1 | 1.147311 | 0.13764 | mRNA |
| ENSRNOG00000050881 | LOC100910581 | -8.49861 | 0.138854 | mRNA |
| ENSRNOG00000021220 | Cpxm1 | -0.543 | 0.145343 | mRNA |
| ENSRNOG00000011316 | Fam167a | -0.58736 | 0.146092 | mRNA |
| ENSRNOG00000058460 | AABR07051551.2 | 2.997358 | 0.146092 | mRNA |
| ENSRNOG00000012095 | Pkia | 0.563063 | 0.14679 | mRNA |
| ENSRNOG00000058562 | AABR07065651.8 | 4.350358 | 0.14679 | mRNA |
| ENSRNOG00000058039 | Acta2 | -0.62962 | 0.14679 | mRNA |
| ENSRNOG00000034191 | Fmo1 | 1.294693 | 0.147833 | mRNA |
| ENSRNOG00000056043 | AABR07065684.1 | 4.52431 | 0.148506 | mRNA |
| ENSRNOG00000037865 | Hormad2 | 2.004539 | 0.148506 | mRNA |
| ENSRNOG00000051158 | Cfb | 1.106041 | 0.154598 | mRNA |
| ENSRNOG00000011483 | S100a9 | 2.268252 | 0.154744 | mRNA |
| ENSRNOG00000014838 | Glipr2 | -0.66702 | 0.157049 | mRNA |
| ENSRNOG00000025184 | Prss35 | -0.5676 | 0.157049 | mRNA |
| ENSRNOG00000009542 | Kcnh5 | 5.419408 | 0.157049 | mRNA |
| ENSRNOG00000048182 | AABR07051684.1 | 2.790412 | 0.157434 | mRNA |
| ENSRNOG00000018715 | Clec10a | 0.923605 | 0.158229 | mRNA |
| ENSRNOG00000016479 | Plekhg4 | -0.87922 | 0.159616 | mRNA |
| ENSRNOG00000049096 | Mcpt8l2 | 2.118707 | 0.159616 | mRNA |
| ENSRNOG00000010805 | Fabp4 | -0.89431 | 0.160244 | mRNA |
| ENSRNOG00000060552 | AABR07065705.5 | 3.518701 | 0.160244 | mRNA |
| ENSRNOG00000013973 | Lcn2 | 2.132398 | 0.166155 | mRNA |
| ENSRNOG00000047641 | AABR07065651.3 | 2.453531 | 0.167117 | mRNA |
| ENSRNOG00000009324 | LOC103692166 | 8.341725 | 0.16757 | mRNA |
| ENSRNOG00000031599 | Sh2d1b | -0.76946 | 0.16767 | mRNA |
| ENSRNOG00000002659 | Ciita | 0.937387 | 0.170507 | mRNA |
| ENSRNOG00000002396 | Serpinb8 | -0.62921 | 0.173372 | mRNA |
| ENSRNOG00000048562 | AC109542.1 | -8.01772 | 0.17346 | mRNA |
| ENSRNOG00000048696 | AABR07065792.2 | 3.494603 | 0.174752 | mRNA |
| ENSRNOG00000021750 | Id1 | 0.835258 | 0.175158 | mRNA |
| ENSRNOG00000003510 | Fmo2 | 1.289828 | 0.176271 | mRNA |
| ENSRNOG00000047321 | Hba-a2 | 1.135545 | 0.179056 | mRNA |
| ENSRNOG00000052224 | Celf6 | 5.417366 | 0.179056 | mRNA |
| ENSRNOG00000057069 | LOC100911272 | 3.320304 | 0.184094 | mRNA |
| ENSRNOG00000022141 | Ctdspl2 | -0.89686 | 0.187765 | mRNA |
| ENSRNOG00000009919 | Acod1 | 2.325014 | 0.194499 | mRNA |
| ENSRNOG00000003888 | Rgs13 | 3.173825 | 0.194499 | mRNA |
| ENSRNOG00000011690 | Rmdn3 | -1.09013 | 0.194499 | mRNA |
| ENSRNOG00000001469 | Eln | -0.72959 | 0.194499 | mRNA |
| ENSRNOG00000049271 | Rad51ap2 | 5.594316 | 0.194583 | mRNA |
| ENSRNOG00000056760 | AABR07065656.8 | 3.330101 | 0.198668 | mRNA |
| ENSRNOG00000038999 | RT1-A1 | 0.549459 | 0.206527 | mRNA |
| ENSRNOG00000013663 | Tmem86a | -0.56886 | 0.206527 | mRNA |
| ENSRNOG00000018251 | Mrc1 | 0.577528 | 0.211263 | mRNA |
| ENSRNOG00000057376 | AABR07051548.2 | 2.403054 | 0.213596 | mRNA |
| ENSRNOG00000013166 | Wnt4 | 1.987902 | 0.21498 | mRNA |
| ENSRNOG00000049299 | AABR07051532.2 | 2.074706 | 0.217128 | mRNA |
| ENSRNOG00000015210 | Ggt6 | -5.39388 | 0.217433 | mRNA |
| ENSRNOG00000018570 | C1qtnf3 | -0.64066 | 0.217433 | mRNA |
| ENSRNOG00000011484 | AABR07049821.1 | 2.260161 | 0.217433 | mRNA |
| ENSRNOG00000046897 | Atrx | -7.85962 | 0.217433 | mRNA |
| ENSRNOG00000009263 | Ifi27 | -0.54584 | 0.217433 | mRNA |
| ENSRNOG00000047535 | AABR07051670.1 | 2.58343 | 0.221978 | mRNA |
| ENSRNOG00000020136 | Tgm1 | 1.953743 | 0.221978 | mRNA |
| ENSRNOG00000054614 | AABR07002779.1 | -1.94221 | 0.221978 | mRNA |
| ENSRNOG00000019718 | Galnt15 | 0.925826 | 0.2248 | mRNA |
| ENSRNOG00000049590 | RT1-M2 | 1.633798 | 0.236204 | mRNA |
| ENSRNOG00000018735 | Cd74 | 0.818281 | 0.246711 | mRNA |
| ENSRNOG00000052455 | LOC100910245 | -4.35597 | 0.249822 | mRNA |
| ENSRNOG00000007625 | B3galt1 | -1.7003 | 0.249822 | mRNA |
| ENSRNOG00000047158 | LOC103689947 | 7.792608 | 0.249822 | mRNA |
| ENSRNOG00000050561 | AABR07065821.1 | 2.023925 | 0.249822 | mRNA |
| ENSRNOG00000042319 | AABR07065814.2 | 3.11031 | 0.250416 | mRNA |
| ENSRNOG00000019120 | Hmgcs2 | 1.619793 | 0.250887 | mRNA |
| ENSRNOG00000016740 | Fam210a | -1.35491 | 0.25423 | mRNA |
| ENSRNOG00000038132 | Vsig4 | 1.972831 | 0.25728 | mRNA |
| ENSRNOG00000015055 | Scg2 | -1.86293 | 0.25728 | mRNA |
| ENSRNOG00000006735 | Cdkn2b | -1.17734 | 0.262024 | mRNA |
| ENSRNOG00000001777 | AABR07034428.1 | -3.58359 | 0.268995 | mRNA |
| ENSRNOG00000032857 | Klk1c9 | -7.61813 | 0.272898 | mRNA |
| ENSRNOG00000002911 | Alb | -6.0725 | 0.273135 | mRNA |
| ENSRNOG00000025164 | Bhlha15 | 2.768744 | 0.273135 | mRNA |
| ENSRNOG00000013042 | Htr1b | 1.342939 | 0.27414 | mRNA |
| ENSRNOG00000006142 | Mymk | -6.43323 | 0.276345 | mRNA |
| ENSRNOG00000048273 | Apod | 0.613545 | 0.28399 | mRNA |
| ENSRNOG00000053402 | Gabrq | 5.419264 | 0.29273 | mRNA |
| ENSRNOG00000002525 | Ptgs2 | -0.78232 | 0.296723 | mRNA |
| ENSRNOG00000021201 | Txnip | 0.440516 | 0.304958 | mRNA |
| ENSRNOG00000016112 | Cd274 | 0.806048 | 0.315027 | mRNA |
| ENSRNOG00000025670 | Shisa3 | 1.143518 | 0.316747 | mRNA |
| ENSRNOG00000055581 | Tarm1 | 5.582959 | 0.316747 | mRNA |
| ENSRNOG00000020625 | Mcpt1 | 1.495888 | 0.317117 | mRNA |
| ENSRNOG00000060654 | AABR07061068.2 | 6.613739 | 0.317602 | mRNA |
| ENSRNOG00000060451 | AABR07065643.1 | 3.526634 | 0.317975 | mRNA |
| ENSRNOG00000012199 | Sox2 | 0.603699 | 0.317975 | mRNA |
| ENSRNOG00000001757 | Tm4sf19 | -0.63685 | 0.317975 | mRNA |
| ENSRNOG00000011913 | Cp | 0.492132 | 0.323963 | mRNA |
| ENSRNOG00000047627 | AABR07002896.1 | 7.49217 | 0.331571 | mRNA |
| ENSRNOG00000053446 | AABR07051665.1 | -3.787 | 0.335852 | mRNA |
| ENSRNOG00000027271 | RGD1359290 | -0.45129 | 0.33691 | mRNA |
| ENSRNOG00000015851 | Nat8b | 7.441596 | 0.33691 | mRNA |
| ENSRNOG00000058105 | Hbb | 1.090018 | 0.340495 | mRNA |
| ENSRNOG00000053122 | Scn1a | -2.19707 | 0.34064 | mRNA |
| ENSRNOG00000027433 | LOC100910708 | -7.35245 | 0.34175 | mRNA |
| ENSRNOG00000025764 | AC128848.1 | 1.622642 | 0.34175 | mRNA |
| ENSRNOG00000043098 | Mt2A | 1.314853 | 0.34175 | mRNA |
| ENSRNOG00000028627 | Hmcn1 | -0.48217 | 0.343976 | mRNA |
| ENSRNOG00000053312 | AABR07065656.4 | 7.398809 | 0.345037 | mRNA |
| ENSRNOG00000045957 | Olr302 | 2.604108 | 0.345605 | mRNA |
| ENSRNOG00000007258 | Sbspon | 0.621413 | 0.349749 | mRNA |
| ENSRNOG00000048951 | LOC100364500 | 1.400207 | 0.351627 | mRNA |
| ENSRNOG00000003620 | Fmo3 | 1.545589 | 0.351894 | mRNA |
| ENSRNOG00000001943 | Retnlg | 1.405966 | 0.354467 | mRNA |
| ENSRNOG00000001212 | Dnmt3l | 3.322352 | 0.358737 | mRNA |
| ENSRNOG00000012721 | Ednra | -1.00724 | 0.358957 | mRNA |
| ENSRNOG00000017248 | Prkag3 | -2.60225 | 0.360597 | mRNA |
| ENSRNOG00000024338 | LOC690276 | 2.930259 | 0.360669 | mRNA |
| ENSRNOG00000020630 | Il9r | 1.66817 | 0.360669 | mRNA |
| ENSRNOG00000049009 | AABR07051532.1 | 3.088866 | 0.361444 | mRNA |
| ENSRNOG00000028708 | Ntsr1 | 2.135377 | 0.367623 | mRNA |
| ENSRNOG00000002451 | Fndc3c1 | -3.42224 | 0.36933 | mRNA |
| ENSRNOG00000050450 | Kcnip2 | -4.34708 | 0.370642 | mRNA |
| ENSRNOG00000038297 | Plekhd1 | -2.23143 | 0.370642 | mRNA |
| ENSRNOG00000004781 | Crmp1 | 0.744162 | 0.370642 | mRNA |
| ENSRNOG00000024726 | Kdm4d | 3.051265 | 0.370642 | mRNA |
| ENSRNOG00000000394 | Srgn | 0.778064 | 0.371313 | mRNA |
| ENSRNOG00000040128 | Cd209a | 5.878558 | 0.377486 | mRNA |
| ENSRNOG00000025406 | Iqgap2 | 0.550414 | 0.380133 | mRNA |
| ENSRNOG00000014081 | Nmur2 | -5.07414 | 0.381546 | mRNA |
| ENSRNOG00000017348 | Il2 | 5.016168 | 0.393818 | mRNA |
| ENSRNOG00000047785 | AABR07065645.2 | 3.451018 | 0.398956 | mRNA |
| ENSRNOG00000002305 | Slc15a2 | 1.185212 | 0.408207 | mRNA |
| ENSRNOG00000008837 | Ass1 | 1.50959 | 0.408207 | mRNA |
| ENSRNOG00000061699 | AABR07061022.3 | 3.238845 | 0.409561 | mRNA |
| ENSRNOG00000061768 | Slc43a3 | 0.966209 | 0.409561 | mRNA |
| ENSRNOG00000057443 | LOC497963 | 2.538167 | 0.411377 | mRNA |
| ENSRNOG00000016875 | Cbx7 | 0.820346 | 0.412882 | mRNA |
| ENSRNOG00000034139 | Lyc2 | 0.50017 | 0.421521 | mRNA |
| ENSRNOG00000005154 | Alkal2 | 3.011798 | 0.424706 | mRNA |
| ENSRNOG00000016812 | Adamts16 | -0.86838 | 0.426732 | mRNA |
| ENSRNOG00000059903 | Thbs3 | -0.49182 | 0.42915 | mRNA |
| ENSRNOG00000039390 | Slc37a2 | -0.4559 | 0.42915 | mRNA |
| ENSRNOG00000050647 | Hspa1b | -0.72094 | 0.435389 | mRNA |
| ENSRNOG00000015986 | Rassf8 | -0.94476 | 0.457957 | mRNA |
| ENSRNOG00000025494 | Nsun7 | 2.57083 | 0.457957 | mRNA |
| ENSRNOG00000021987 | Tnfrsf17 | 2.215048 | 0.462976 | mRNA |
| ENSRNOG00000050024 | Ms4a4a | 0.499091 | 0.462976 | mRNA |
| ENSRNOG00000006333 | AABR07065789.1 | 1.919066 | 0.464559 | mRNA |
| ENSRNOG00000058645 | Tnc | -0.43366 | 0.470804 | mRNA |
| ENSRNOG00000016120 | Tlx1 | -2.27141 | 0.470804 | mRNA |
| ENSRNOG00000021644 | Slc15a3 | -0.55153 | 0.471657 | mRNA |
| ENSRNOG00000008184 | Esrp1 | -5.06557 | 0.474704 | mRNA |
| ENSRNOG00000008141 | Nppb | -1.93777 | 0.476532 | mRNA |
| ENSRNOG00000014350 | Ccn1 | -0.4756 | 0.48087 | mRNA |
| ENSRNOG00000020246 | Myl9 | -0.54383 | 0.49073 | mRNA |
| ENSRNOG00000021945 | Ccdc184 | 2.38482 | 0.49073 |  |
| ENSRNOG00000042978 | Ncald | -0.52073 | 0.49073 |  |
