# Supplementary material for: Biological characteristics of tissue engineered-nerve grafts enhancing peripheral nerve regeneration
Source: Stem Cell Res Ther. 2024 Jul 18;15:215. doi: 10.1186/s13287-024-03827-9 (PMC11256578; doi:10.1186/s13287-024-03827-9)
Supplement: Supplementary file 2 — Additional file 2. [file 13287_2024_3827_MOESM2_ESM.docx]

**Supplementary Table2 Differentially expressed genes of lncRNA (ASC vs ANA)**

| Gene ID | Gene name | Log2(Fold Change) | padj | Type |
| --- | --- | --- | --- | --- |
|  |  |  |  |  |
| ENSRNOG00000023257 | Adamts9 | 11.34509206 | 1.72E-09 | lncRNA |
| ENSRNOG00000047746 | AABR07000398.1 | -11.54061418 | 2.98E-09 | lncRNA |
| ENSRNOG00000055700 | AC096473.3 | -10.45305794 | 3.12E-07 | lncRNA |
| ENSRNOG00000057054 | LOC103690118 | 8.802519197 | 5.35E-05 | lncRNA |
| ENSRNOG00000056984 | Baz2b | 5.930005965 | 0.000135 | lncRNA |
| ENSRNOG00000058834 | LOC103692471 | -8.279183314 | 0.000253 | lncRNA |
| XLOC_016985 | XLOC_016985 | -13.15280927 | 0.000283 | lncRNA |
| XLOC_004169 | XLOC_004169 | 12.313594 | 0.000354 | lncRNA |
| ENSRNOG00000061691 | LOC102550455 | 8.993151927 | 0.000699 | lncRNA |
| ENSRNOG00000047573 | Ttc39a | -11.53937026 | 0.001018 | lncRNA |
| ENSRNOG00000010057 | Clec4n | 8.03080975 | 0.001296 | lncRNA |
| ENSRNOG00000049829 | AABR07060872.1 | 2.915839755 | 0.001374 | lncRNA |
| XLOC_014975 | XLOC_014975 | 11.327318 | 0.001555 | lncRNA |
| XLOC_028912 | XLOC_028912 | -10.80994043 | 0.003131 | lncRNA |
| ENSRNOG00000049908 | AABR07037436.1 | 5.609857202 | 0.003131 | lncRNA |
| ENSRNOG00000012270 | Med26 | -10.69747259 | 0.003371 | lncRNA |
| XLOC_001428 | XLOC_001428 | -10.59109809 | 0.003572 | lncRNA |
| ENSRNOG00000061122 | LOC102551683 | 7.29597888 | 0.004577 | lncRNA |
| ENSRNOG00000050000 | AABR07034739.1 | 10.4926373 | 0.004767 | lncRNA |
| ENSRNOG00000047573 | Ttc39a | -10.20218273 | 0.005386 | lncRNA |
| ENSRNOG00000051941 | Fam78b | -9.819530793 | 0.007691 | lncRNA |
| XLOC_016770 | XLOC_016770 | -9.350633707 | 0.011097 | lncRNA |
| ENSRNOG00000039754 | Rab7b | -9.3611395 | 0.011167 | lncRNA |
| XLOC_001428 | XLOC_001428 | -8.331754445 | 0.013821 | lncRNA |
| XLOC_001428 | XLOC_001428 | -9.884668601 | 0.013823 | lncRNA |
| ENSRNOG00000047933 | LOC103690164 | -10.8328 | 1.59E-23 | mRNA |
| ENSRNOG00000050000 | AABR07034739.1 | 3.570738 | 1.59E-23 | mRNA |
| ENSRNOG00000049829 | AABR07060872.1 | 2.667252 | 1.59E-17 | mRNA |
| ENSRNOG00000046246 | AABR07015881.1 | 10.03406 | 2.07E-16 | mRNA |
| ENSRNOG00000034190 | Ighm | 2.676859 | 4.62E-15 | mRNA |
| ENSRNOG00000043451 | Spp1 | -1.87335 | 5.70E-09 | mRNA |
| ENSRNOG00000049315 | AABR07065827.1 | 3.679535 | 1.61E-08 | mRNA |
| ENSRNOG00000010906 | Ccl5 | 3.408351 | 3.14E-08 | mRNA |
| ENSRNOG00000003666 | Jchain | 1.871993 | 6.57E-08 | mRNA |
| ENSRNOG00000030332 | AABR07065670.1 | 5.799595 | 7.34E-08 | mRNA |
| ENSRNOG00000030187 | Mmp12 | -1.57661 | 2.55E-06 | mRNA |
| ENSRNOG00000048167 | AABR07051551.1 | 3.534029 | 2.74E-06 | mRNA |
| ENSRNOG00000052619 | AABR07065680.1 | 5.948768 | 3.09E-06 | mRNA |
| ENSRNOG00000048425 | AABR07065776.3 | 3.823964 | 8.36E-06 | mRNA |
| ENSRNOG00000000562 | Prf1 | 2.904831 | 1.17E-05 | mRNA |
| ENSRNOG00000048982 | Calcoco1 | 12.51881 | 1.17E-05 | mRNA |
| ENSRNOG00000057755 | NEWGENE_1304700 | -12.487 | 1.17E-05 | mRNA |
| ENSRNOG00000033376 | AABR07065782.1 | 4.775242 | 2.43E-05 | mRNA |
| ENSRNOG00000049814 | LOC100910882 | 12.58675 | 2.43E-05 | mRNA |
| ENSRNOG00000022009 | Mzb1 | 2.446471 | 2.72E-05 | mRNA |
| ENSRNOG00000054459 | Mboat7 | -11.6128 | 3.74E-05 | mRNA |
| ENSRNOG00000051600 | Ly49i4 | 3.71099 | 4.23E-05 | mRNA |
| ENSRNOG00000048402 | AABR07065625.2 | 3.056368 | 9.65E-05 | mRNA |
| ENSRNOG00000056285 | AABR07065693.3 | 3.022611 | 0.000103 | mRNA |
| ENSRNOG00000053103 | AABR07065705.2 | 4.008543 | 0.000121 | mRNA |
| ENSRNOG00000047103 | AABR07065656.1 | 5.3094 | 0.000122 | mRNA |
| ENSRNOG00000059953 | AABR07065656.10 | 10.22422 | 0.00017 | mRNA |
| ENSRNOG00000012181 | Lpl | -1.2318 | 0.000312 | mRNA |
| ENSRNOG00000046572 | AABR07065651.2 | 4.512583 | 0.000393 | mRNA |
| ENSRNOG00000059121 | AABR07065714.1 | 3.642481 | 0.000418 | mRNA |
| ENSRNOG00000042193 | AABR07034729.1 | 2.437486 | 0.000517 | mRNA |
| ENSRNOG00000048458 | AABR07065705.1 | 8.752156 | 0.000531 | mRNA |
| ENSRNOG00000047415 | AABR07060788.1 | 7.199845 | 0.000576 | mRNA |
| ENSRNOG00000042083 | AABR07051562.1 | 2.647699 | 0.000638 | mRNA |
| ENSRNOG00000011500 | Pou2af1 | 2.037508 | 0.000638 | mRNA |
| ENSRNOG00000057643 | Klri2 | 5.609192 | 0.000646 | mRNA |
| ENSRNOG00000047790 | B9d1 | 9.658091 | 0.000646 | mRNA |
| ENSRNOG00000036698 | Nploc4 | -0.95681 | 0.0007 | mRNA |
| ENSRNOG00000010661 | Gzmk | 2.292914 | 0.000726 | mRNA |
| ENSRNOG00000052069 | AABR07065673.1 | 3.510373 | 0.000778 | mRNA |
| ENSRNOG00000050898 | AABR07034730.2 | 3.207589 | 0.000836 | mRNA |
| ENSRNOG00000054513 | LOC103693776 | 8.717681 | 0.000836 | mRNA |
| ENSRNOG00000047940 | LOC103694864 | -9.30174 | 0.0009 | mRNA |
| ENSRNOG00000059176 | AABR07060963.2 | 7.51236 | 0.0009 | mRNA |
| ENSRNOG00000061699 | AABR07061022.3 | 4.28036 | 0.0009 | mRNA |
| ENSRNOG00000053719 | AABR07065656.5 | 4.635563 | 0.000929 | mRNA |
| ENSRNOG00000050158 | LOC100911692 | -9.3627 | 0.000929 | mRNA |
| ENSRNOG00000051860 | AC114343.1 | -2.66524 | 0.000931 | mRNA |
| ENSRNOG00000051661 | Ly49s5 | 4.023729 | 0.001418 | mRNA |
| ENSRNOG00000056179 | AABR07061072.1 | 4.798792 | 0.001452 | mRNA |
| ENSRNOG00000052467 | Klrc3 | 3.403958 | 0.001465 | mRNA |
| ENSRNOG00000053891 | AABR07018078.1 | 1.75 | 0.001465 | mRNA |
| ENSRNOG00000059447 | AC109901.2 | 8.991408 | 0.001589 | mRNA |
| ENSRNOG00000017749 | Nkg7 | 2.505459 | 0.001644 | mRNA |
| ENSRNOG00000058824 | AABR07065789.3 | 4.464123 | 0.001724 | mRNA |
| ENSRNOG00000054828 | AABR07051708.1 | 3.131391 | 0.001752 | mRNA |
| ENSRNOG00000031848 | AABR07065651.1 | 3.339461 | 0.001796 | mRNA |
| ENSRNOG00000060552 | AABR07065705.5 | 4.949241 | 0.001883 | mRNA |
| ENSRNOG00000004402 | Lpgat1 | 0.849303 | 0.002179 | mRNA |
| ENSRNOG00000007200 | Ikzf3 | 1.708551 | 0.002469 | mRNA |
| ENSRNOG00000047350 | Gns | -1.12944 | 0.002547 | mRNA |
| ENSRNOG00000051694 | AABR07065651.6 | 4.413063 | 0.002701 | mRNA |
| ENSRNOG00000030235 | AABR07065823.2 | 2.343343 | 0.002701 | mRNA |
| ENSRNOG00000050792 | Tnfaip6 | -0.9816 | 0.003323 | mRNA |
| ENSRNOG00000027466 | Cd27 | 2.283917 | 0.003461 | mRNA |
| ENSRNOG00000001959 | Mx1 | 1.782066 | 0.003531 | mRNA |
| ENSRNOG00000047571 | RGD1563231 | 2.933954 | 0.003642 | mRNA |
| ENSRNOG00000026605 | Ifi27l2b | 1.602873 | 0.003923 | mRNA |
| ENSRNOG00000051768 | AABR07065693.2 | 3.304825 | 0.00404 | mRNA |
| ENSRNOG00000056162 | AC111885.1 | -2.548 | 0.00423 | mRNA |
| ENSRNOG00000057220 | AABR07060792.1 | 4.476546 | 0.004576 | mRNA |
| ENSRNOG00000058460 | AABR07051551.2 | 2.602724 | 0.004757 | mRNA |
| ENSRNOG00000057421 | AABR07065699.3 | 3.173983 | 0.004833 | mRNA |
| ENSRNOG00000057092 | Slfn4 | 1.387056 | 0.005216 | mRNA |
| ENSRNOG00000006314 | Zbp1 | 1.848359 | 0.005387 | mRNA |
| ENSRNOG00000053527 | AABR07034736.1 | 5.208912 | 0.005819 | mRNA |
| ENSRNOG00000018711 | Ppcdc | -8.17348 | 0.006726 | mRNA |
| ENSRNOG00000060654 | AABR07061068.2 | 9.691317 | 0.006861 | mRNA |
| ENSRNOG00000032970 | Gpr174 | 2.011308 | 0.007152 | mRNA |
| ENSRNOG00000059910 | AABR07051689.1 | 2.466345 | 0.007177 | mRNA |
| ENSRNOG00000048159 | AABR07065778.3 | 2.659468 | 0.007181 | mRNA |
| ENSRNOG00000045682 | AABR07060992.1 | 9.722752 | 0.00727 | mRNA |
| ENSRNOG00000043061 | Siah3 | 6.487362 | 0.00727 | mRNA |
| XLOC_010293 | XLOC_010293 | 1.702651 | 0.007375 | mRNA |
| ENSRNOG00000058590 | AABR07034739.2 | 1.878019 | 0.007484 | mRNA |
| ENSRNOG00000060246 | Klrd1 | 2.788805 | 0.007484 | mRNA |
| ENSRNOG00000007664 | Tnfrsf13c | 2.449319 | 0.007497 | mRNA |
| ENSRNOG00000023465 | Depp1 | -1.45487 | 0.007567 | mRNA |
| ENSRNOG00000050066 | AABR07051583.1 | 3.052039 | 0.009594 | mRNA |
| ENSRNOG00000042140 | Eomes | 2.560294 | 0.009841 | mRNA |
| ENSRNOG00000046449 | LOC100912228 | 6.1734 | 0.010162 | mRNA |
| ENSRNOG00000039744 | RT1-CE4 | 0.995382 | 0.011145 | mRNA |
| ENSRNOG00000060898 | LOC100910418 | -12.0728 | 0.011654 | mRNA |
| ENSRNOG00000053312 | AABR07065656.4 | 7.869956 | 0.011717 | mRNA |
| ENSRNOG00000048636 | Il2rb | 1.846205 | 0.011846 | mRNA |
| ENSRNOG00000021334 | Lag3 | 2.393459 | 0.011846 | mRNA |
| ENSRNOG00000000322 | Atg5 | 1.035271 | 0.01229 | mRNA |
| ENSRNOG00000056290 | AABR07061052.1 | 3.070437 | 0.012309 | mRNA |
| ENSRNOG00000003977 | Dusp1 | -0.8183 | 0.012322 | mRNA |
| ENSRNOG00000017557 | LOC100362216 | 6.281962 | 0.012486 | mRNA |
| ENSRNOG00000043155 | AABR07051533.2 | 3.796406 | 0.013134 | mRNA |
| ENSRNOG00000018238 | Nuggc | 3.473852 | 0.013558 | mRNA |
| ENSRNOG00000006263 | Sh2d1a | 2.567642 | 0.013607 | mRNA |
| ENSRNOG00000045560 | Gvin1 | 1.488466 | 0.014792 | mRNA |
| ENSRNOG00000047658 | AABR07034718.1 | 6.710093 | 0.014882 | mRNA |
| ENSRNOG00000007811 | Klrb1c | 2.623223 | 0.016195 | mRNA |
| ENSRNOG00000045599 | AABR07027450.1 | 6.307726 | 0.016386 | mRNA |
| ENSRNOG00000030530 | Gzmm | 2.317147 | 0.016485 | mRNA |
| ENSRNOG00000046011 | AABR07065645.1 | 3.060466 | 0.016648 | mRNA |
| ENSRNOG00000007545 | Angptl4 | -0.98521 | 0.017002 | mRNA |
| ENSRNOG00000014771 | Wfdc8 | -6.25185 | 0.017122 | mRNA |
| ENSRNOG00000017414 | Irf7 | 1.477201 | 0.017122 | mRNA |
| ENSRNOG00000042197 | AABR07065792.1 | 3.820333 | 0.01779 | mRNA |
| ENSRNOG00000049243 | AABR07065656.3 | 3.526633 | 0.017999 | mRNA |
| ENSRNOG00000030812 | AABR07065781.1 | 2.00238 | 0.018077 | mRNA |
| ENSRNOG00000009427 | Tbx21 | 2.048716 | 0.018207 | mRNA |
| ENSRNOG00000013304 | Arg1 | -1.2411 | 0.018609 | mRNA |
| ENSRNOG00000048070 | AABR07051563.1 | 2.666066 | 0.020387 | mRNA |
| ENSRNOG00000053228 | AABR07061036.1 | 3.693758 | 0.020462 | mRNA |
| ENSRNOG00000004247 | Nhp2 | -11.0405 | 0.020462 | mRNA |
| ENSRNOG00000048182 | AABR07051684.1 | 3.553086 | 0.020462 | mRNA |
| ENSRNOG00000055375 | AABR07065837.1 | 3.024093 | 0.020462 | mRNA |
| ENSRNOG00000001757 | Tm4sf19 | -0.96138 | 0.020499 | mRNA |
| ENSRNOG00000047782 | RGD1309808 | 1.744398 | 0.021572 | mRNA |
| ENSRNOG00000057548 | AABR07051741.1 | 2.651469 | 0.022069 | mRNA |
| ENSRNOG00000049895 | LOC100910143 | -10.6089 | 0.022069 | mRNA |
| ENSRNOG00000057165 | AABR07034730.3 | 3.165614 | 0.022305 | mRNA |
| ENSRNOG00000059120 | AABR07011031.1 | -3.8816 | 0.022422 | mRNA |
| ENSRNOG00000053337 | Ly49s6 | 2.718192 | 0.022422 | mRNA |
| ENSRNOG00000059679 | LOC103692165 | 10.90685 | 0.023345 | mRNA |
| ENSRNOG00000046883 | Mydgf | -0.92907 | 0.023345 | mRNA |
| ENSRNOG00000059199 | AABR07065705.4 | 4.888611 | 0.023345 | mRNA |
| ENSRNOG00000029001 | RT1-S2 | 7.173333 | 0.024899 | mRNA |
| ENSRNOG00000055909 | Apoa4 | 3.402535 | 0.025147 | mRNA |
| ENSRNOG00000039955 | Samd3 | 2.857721 | 0.02544 | mRNA |
| ENSRNOG00000014312 | AC109737.1 | 1.79962 | 0.02544 | mRNA |
| ENSRNOG00000022021 | Spag6 | 3.248439 | 0.026162 | mRNA |
| ENSRNOG00000018458 | Ncr1 | 2.554743 | 0.026497 | mRNA |
| ENSRNOG00000046968 | Nol8 | 2.031241 | 0.026497 | mRNA |
| ENSRNOG00000012199 | Sox2 | 0.751602 | 0.026497 | mRNA |
| ENSRNOG00000010057 | Clec4n | 2.771814 | 0.026512 | mRNA |
| ENSRNOG00000045973 | Gzmbl2 | 2.458983 | 0.026589 | mRNA |
| ENSRNOG00000025925 | Txk | 1.935418 | 0.026589 | mRNA |
| ENSRNOG00000048017 | AABR07056026.1 | 4.221178 | 0.026589 | mRNA |
| ENSRNOG00000016995 | Zap70 | 1.713216 | 0.027224 | mRNA |
| ENSRNOG00000028250 | Lax1 | 1.944768 | 0.028203 | mRNA |
| ENSRNOG00000023068 | Cd5l | -1.38398 | 0.028838 | mRNA |
| ENSRNOG00000029762 | Acr | -6.10592 | 0.029217 | mRNA |
| ENSRNOG00000024338 | LOC690276 | 3.213756 | 0.029217 | mRNA |
| ENSRNOG00000059007 | AABR07061005.2 | 3.816677 | 0.03043 | mRNA |
| ENSRNOG00000056043 | AABR07065684.1 | 3.598574 | 0.030638 | mRNA |
| ENSRNOG00000025164 | Bhlha15 | 2.784587 | 0.030722 | mRNA |
| ENSRNOG00000031743 | Gbp2 | 1.090557 | 0.032573 | mRNA |
| ENSRNOG00000027096 | Ctsw | 1.630933 | 0.033081 | mRNA |
| ENSRNOG00000045677 | Nlrp6 | -10.0125 | 0.034903 | mRNA |
| ENSRNOG00000050102 | AABR07065699.1 | 3.668105 | 0.036164 | mRNA |
| ENSRNOG00000011917 | Cd79b | 1.837844 | 0.037615 | mRNA |
| ENSRNOG00000053487 | AABR07065877.1 | 8.459448 | 0.039541 | mRNA |
| ENSRNOG00000058714 | Klre1 | 4.629269 | 0.039541 | mRNA |
| ENSRNOG00000055196 | Klrc1 | 2.573011 | 0.039541 | mRNA |
| ENSRNOG00000047535 | AABR07051670.1 | 2.079359 | 0.039906 | mRNA |
| ENSRNOG00000040108 | RGD1565355 | -0.74075 | 0.039906 | mRNA |
| ENSRNOG00000007310 | Klrb1b | 1.977537 | 0.043194 | mRNA |
| ENSRNOG00000017897 | Adam8 | -0.88704 | 0.045963 | mRNA |
| ENSRNOG00000052512 | Vps37c | -9.44877 | 0.046248 | mRNA |
| ENSRNOG00000016753 | Slc14a1 | 1.844774 | 0.04739 | mRNA |
| ENSRNOG00000013663 | Tmem86a | -0.73978 | 0.048801 | mRNA |
| ENSRNOG00000042319 | AABR07065814.2 | 2.604093 | 0.048822 | mRNA |
| ENSRNOG00000051739 | AABR07061001.1 | 2.279948 | 0.049407 | mRNA |
| ENSRNOG00000006046 | Gdf7 | 3.998801 | 0.049458 | mRNA |
