# Supplementary material for: Biological characteristics of tissue engineered-nerve grafts enhancing peripheral nerve regeneration
Source: Stem Cell Res Ther. 2024 Jul 18;15:215. doi: 10.1186/s13287-024-03827-9 (PMC11256578; doi:10.1186/s13287-024-03827-9)
Supplement: Supplementary file 3 — Additional file 3. [file 13287_2024_3827_MOESM3_ESM.docx]

**Supplementary Table 3 Differentially expressed genes of lncRNA (BM vs ASC)**

| Gene ID | Gene name | Log2(Fold Change) | padj | Type |
| --- | --- | --- | --- | --- |
| ENSRNOG00000004452 | Aqp12a | -13.2104 | 2.98E-21 | lncRNA |
| ENSRNOG00000004452 | Aqp12a | 13.12303 | 1.32E-20 | lncRNA |
| ENSRNOG00000047746 | AABR07000398.1 | 10.63997 | 3.47E-18 | lncRNA |
| XLOC_028844 | XLOC_028844 | -10.2314 | 3.23E-06 | lncRNA |
| XLOC_001428 | XLOC_001428 | 9.889536 | 5.81E-06 | lncRNA |
| XLOC_012648 | XLOC_012648 | -14.864 | 1.21E-05 | lncRNA |
| ENSRNOG00000011882 | Gab2 | -13.6109 | 8.00E-05 | lncRNA |
| XLOC_014975 | XLOC_014975 | -11.3601 | 0.002169 | lncRNA |
| ENSRNOG00000028659 | Szt2 | -8.72388 | 0.004169 | lncRNA |
| ENSRNOG00000009075 | Trim13 | -10.8328 | 0.004239 | lncRNA |
| ENSRNOG00000058316 | LOC686087 | 10.66482 | 0.004611 | lncRNA |
| ENSRNOG00000034139 | Lyc2 | -9.2205 | 0.007147 | lncRNA |
| XLOC_027339 | XLOC_027339 | -10.5737 | 0.00846 | lncRNA |
| XLOC_018506 | XLOC_018506 | 9.144951 | 0.009049 | lncRNA |
| ENSRNOG00000047746 | AABR07000398.1 | -15.5579 | 0.009576 | lncRNA |
| ENSRNOG00000032605 | AC118412.1 | 8.827236 | 0.010689 | lncRNA |
| XLOC_016770 | XLOC_016770 | 9.222068 | 0.016404 | lncRNA |
| ENSRNOG00000059087 | LOC102546889 | 9.136475 | 0.018257 | lncRNA |
| XLOC_001428 | XLOC_001428 | 9.047372 | 0.01982 | lncRNA |
| XLOC_015314 | XLOC_015314 | 9.411736 | 0.020401 | lncRNA |
| XLOC_015126 | XLOC_015126 | -13.3995 | 0.020621 | lncRNA |
| ENSRNOG00000019596 | Smg9 | 8.217058 | 0.021111 | lncRNA |
| XLOC_020471 | XLOC_020471 | -8.90889 | 0.030403 | lncRNA |
| ENSRNOG00000053185 | AABR07006889.1 | -9.44028 | 0.031721 | lncRNA |
| XLOC_009888 | XLOC_009888 | -8.44372 | 0.034077 | lncRNA |
| ENSRNOG00000011508 | Zc3h12b | -6.57096 | 0.047374 | lncRNA |
| ENSRNOG00000057755 | NEWGENE_1304700 | 13.01099 | 2.04E-58 | mRNA |
| ENSRNOG00000054459 | Mboat7 | 11.96654 | 2.24E-33 | mRNA |
| ENSRNOG00000045831 | Lmod2 | -10.7757 | 6.15E-21 | mRNA |
| ENSRNOG00000047940 | LOC103694864 | 9.981144 | 3.17E-15 | mRNA |
| ENSRNOG00000012721 | Ednra | -1.7885 | 1.24E-13 | mRNA |
| ENSRNOG00000048935 | Tmem45a | -11.004 | 1.77E-13 | mRNA |
| ENSRNOG00000017414 | Irf7 | -2.2962 | 1.43E-09 | mRNA |
| ENSRNOG00000001959 | Mx1 | -2.24539 | 2.21E-08 | mRNA |
| ENSRNOG00000010906 | Ccl5 | -3.28983 | 2.97E-07 | mRNA |
| ENSRNOG00000052925 | NEWGENE_621351 | -15.4453 | 1.06E-06 | mRNA |
| ENSRNOG00000053228 | AABR07061036.1 | -9.96431 | 1.06E-06 | mRNA |
| ENSRNOG00000033376 | AABR07065782.1 | -4.97743 | 1.12E-06 | mRNA |
| ENSRNOG00000043107 | LOC103693563 | -5.40093 | 1.73E-06 | mRNA |
| ENSRNOG00000050994 | Cttn | 13.93643 | 2.96E-06 | mRNA |
| ENSRNOG00000001963 | Mx2 | -1.79026 | 6.88E-06 | mRNA |
| ENSRNOG00000048425 | AABR07065776.3 | -3.8604 | 7.44E-06 | mRNA |
| ENSRNOG00000053891 | AABR07018078.1 | -2.04341 | 1.16E-05 | mRNA |
| ENSRNOG00000057092 | Slfn4 | -1.7627 | 1.32E-05 | mRNA |
| ENSRNOG00000018911 | Pfkfb3 | 1.418887 | 1.82E-05 | mRNA |
| ENSRNOG00000053772 | Klra22 | -4.72085 | 2.51E-05 | mRNA |
| ENSRNOG00000026605 | Ifi27l2b | -1.92941 | 3.43E-05 | mRNA |
| ENSRNOG00000030812 | AABR07065781.1 | -2.74312 | 5.88E-05 | mRNA |
| ENSRNOG00000022141 | Ctdspl2 | -1.11197 | 7.41E-05 | mRNA |
| ENSRNOG00000000562 | Prf1 | -2.80394 | 0.000113 | mRNA |
| ENSRNOG00000051600 | Ly49i4 | -3.8615 | 0.000121 | mRNA |
| ENSRNOG00000007811 | Klrb1c | -3.5609 | 0.000138 | mRNA |
| ENSRNOG00000010661 | Gzmk | -2.62675 | 0.000246 | mRNA |
| ENSRNOG00000047933 | LOC103690164 | 10.27524 | 0.000335 | mRNA |
| ENSRNOG00000030530 | Gzmm | -2.91855 | 0.000335 | mRNA |
| ENSRNOG00000017749 | Nkg7 | -2.85906 | 0.00035 | mRNA |
| ENSRNOG00000053337 | Ly49s6 | -3.30071 | 0.000384 | mRNA |
| ENSRNOG00000060898 | LOC100910418 | 11.50691 | 0.000409 | mRNA |
| ENSRNOG00000042140 | Eomes | -3.16415 | 0.000415 | mRNA |
| ENSRNOG00000021802 | Isg15 | -2.06174 | 0.000427 | mRNA |
| ENSRNOG00000033017 | LOC100910669 | -3.36763 | 0.000601 | mRNA |
| ENSRNOG00000050881 | LOC100910581 | -9.44027 | 0.001008 | mRNA |
| ENSRNOG00000027466 | Cd27 | -2.41323 | 0.001163 | mRNA |
| ENSRNOG00000001777 | AABR07034428.1 | -4.91131 | 0.001163 | mRNA |
| ENSRNOG00000059176 | AABR07060963.2 | -7.53789 | 0.0013 | mRNA |
| ENSRNOG00000023969 | Herc6 | -1.22849 | 0.001327 | mRNA |
| ENSRNOG00000008210 | Ky | -9.65559 | 0.001488 | mRNA |
| ENSRNOG00000001187 | Oasl | -1.64079 | 0.001576 | mRNA |
| ENSRNOG00000042905 | RT1-T24-4 | -1.27583 | 0.001607 | mRNA |
| ENSRNOG00000005269 | Srl | -3.60096 | 0.001677 | mRNA |
| ENSRNOG00000011557 | S100a8 | 3.222672 | 0.001784 | mRNA |
| ENSRNOG00000006930 | Casq1 | -3.11663 | 0.002381 | mRNA |
| ENSRNOG00000002916 | Car4 | 9.263742 | 0.002895 | mRNA |
| ENSRNOG00000011483 | S100a9 | 3.183197 | 0.003847 | mRNA |
| ENSRNOG00000017897 | Adam8 | 1.031432 | 0.004084 | mRNA |
| ENSRNOG00000036698 | Nploc4 | 0.875824 | 0.004084 | mRNA |
| ENSRNOG00000001369 | Oas1a | -1.53916 | 0.004519 | mRNA |
| ENSRNOG00000013532 | Pgam2 | -4.54908 | 0.005041 | mRNA |
| ENSRNOG00000002843 | Cxcl6 | 2.378608 | 0.005085 | mRNA |
| ENSRNOG00000002964 | Xcl1 | -3.30793 | 0.005551 | mRNA |
| ENSRNOG00000037198 | Usp18 | -1.05604 | 0.006428 | mRNA |
| ENSRNOG00000057069 | LOC100911272 | 4.165018 | 0.006458 | mRNA |
| ENSRNOG00000032857 | Klk1c9 | -8.25754 | 0.007512 | mRNA |
| ENSRNOG00000045924 | RT1-T24-3 | -1.36804 | 0.007729 | mRNA |
| ENSRNOG00000019183 | Alox15 | 2.772029 | 0.007729 | mRNA |
| ENSRNOG00000014378 | Il1r2 | 2.482308 | 0.008125 | mRNA |
| ENSRNOG00000033844 | LOC100912195 | -6.38996 | 0.008125 | mRNA |
| ENSRNOG00000043451 | Spp1 | 0.9566 | 0.008518 | mRNA |
| ENSRNOG00000062232 | Mcc | -6.52371 | 0.008925 | mRNA |
| ENSRNOG00000006314 | Zbp1 | -1.91636 | 0.010275 | mRNA |
| ENSRNOG00000060246 | Klrd1 | -2.80015 | 0.011236 | mRNA |
| ENSRNOG00000051661 | Ly49s5 | -4.46567 | 0.013982 | mRNA |
| ENSRNOG00000013973 | Lcn2 | 2.756935 | 0.014452 | mRNA |
| ENSRNOG00000019278 | Fsd2 | -4.74328 | 0.01545 | mRNA |
| ENSRNOG00000022839 | Ifit3 | -1.3345 | 0.01545 | mRNA |
| ENSRNOG00000008310 | Mpo | 3.927701 | 0.016039 | mRNA |
| ENSRNOG00000059097 | AABR07025140.1 | -0.97637 | 0.017954 | mRNA |
| ENSRNOG00000027096 | Ctsw | -1.81537 | 0.018169 | mRNA |
| ENSRNOG00000026110 | Scml4 | -2.45964 | 0.018169 | mRNA |
| ENSRNOG00000048167 | AABR07051551.1 | -2.25505 | 0.018169 | mRNA |
| ENSRNOG00000029286 | Hbe1 | -4.70049 | 0.018169 | mRNA |
| ENSRNOG00000029658 | Rnf213 | -0.89142 | 0.019108 | mRNA |
| ENSRNOG00000029386 | RT1-N2 | -1.34821 | 0.019622 | mRNA |
| ENSRNOG00000050275 | LOC100912399 | -12.3514 | 0.019906 | mRNA |
| ENSRNOG00000050864 | Cpne1 | 12.18687 | 0.019974 | mRNA |
| ENSRNOG00000010663 | Col6a5 | 2.668872 | 0.020247 | mRNA |
| ENSRNOG00000040108 | RGD1565355 | 0.917496 | 0.021098 | mRNA |
| ENSRNOG00000043288 | Smim13 | 2.413361 | 0.021098 | mRNA |
| ENSRNOG00000010347 | Styk1 | -2.69359 | 0.021894 | mRNA |
| ENSRNOG00000061739 | Klrk1 | -1.21461 | 0.022465 | mRNA |
| ENSRNOG00000008376 | LOC100909595 | 1.139122 | 0.023093 | mRNA |
| ENSRNOG00000028814 | Oasl2 | -1.01308 | 0.023093 | mRNA |
| ENSRNOG00000007545 | Angptl4 | 1.079228 | 0.023678 | mRNA |
| ENSRNOG00000059762 | AABR07003833.1 | -3.87706 | 0.024956 | mRNA |
| ENSRNOG00000037645 | Tceal7 | -4.78229 | 0.024956 | mRNA |
| ENSRNOG00000049282 | Oas2 | -1.6612 | 0.02538 | mRNA |
| ENSRNOG00000046452 | Fcgr2b | 0.900836 | 0.025859 | mRNA |
| ENSRNOG00000006224 | Klhl31 | -3.14153 | 0.027266 | mRNA |
| ENSRNOG00000047746 | AABR07000398.1 | -0.92327 | 0.027266 | mRNA |
| ENSRNOG00000052619 | AABR07065680.1 | -3.91337 | 0.027266 | mRNA |
| ENSRNOG00000046276 | Myh3 | -3.1179 | 0.027266 | mRNA |
| ENSRNOG00000059900 | Bst2 | -1.14188 | 0.029655 | mRNA |
| ENSRNOG00000014118 | Klkb1 | -8.45604 | 0.035487 | mRNA |
| ENSRNOG00000059207 | Oas3 | -1.59305 | 0.036548 | mRNA |
| ENSRNOG00000047706 | LOC103690108 | 6.003355 | 0.037217 | mRNA |
| ENSRNOG00000055450 | Ckmt2 | -6.43616 | 0.037325 | mRNA |
| ENSRNOG00000049895 | LOC100910143 | 10.7474 | 0.037325 | mRNA |
| ENSRNOG00000059953 | AABR07065656.10 | -5.09748 | 0.037325 | mRNA |
| ENSRNOG00000023614 | Hsh2d | -1.58564 | 0.039681 | mRNA |
| ENSRNOG00000009427 | Tbx21 | -2.23788 | 0.041463 | mRNA |
| ENSRNOG00000056947 | AABR07025272.1 | -1.03455 | 0.044018 | mRNA |
| ENSRNOG00000018458 | Ncr1 | -2.63799 | 0.045824 | mRNA |
| ENSRNOG00000061091 | AABR07045071.1 | -7.29409 | 0.047187 | mRNA |
| ENSRNOG00000023943 | AABR07013651.1 | -2.76846 | 0.048763 | mRNA |
